# Integrated analysis of single-cell and bulk transcriptomics reveals the prognostic value and underlying mechanisms of crotonylation in ovarian cancer

**DOI:** 10.3389/fimmu.2025.1596080

**Published:** 2025-09-10

**Authors:** Xiaofeng Li, Weimin Wu, Jie Tao, Xiaoqing Guo

**Affiliations:** ^1^ Department of Gynecology, Shanghai First Maternity and Infant Hospital, School of Medicine, Tongji University, Shanghai, China; ^2^ Shanghai Key Laboratory of Maternal Fetal Medicine, Shanghai Institute of Maternal-Fetal Medicine and Gynecologic Oncology, Shanghai First Maternity and Infant Hospital, School of Medicine, Tongji University, Shanghai, China

**Keywords:** ovarian cancer, crotonylation, tumor progression, tumor microenvironment, prognostic value

## Abstract

**Background:**

Ovarian cancer remains the deadliest gynecological malignancy with 5-year survival rates below 40% due to frequent recurrence and chemoresistance. Aberrant crotonylation, a type of epigenetic modification, has been implicated in the proliferation, metastasis, and immune evasion of various cancers. However, its role in the ovarian cancer microenvironment and clinical outcomes remains unexplored. The aim of this study was to develop a prognostic model for ovarian cancer on the basis of crotonylation and to investigate the underlying mechanisms and potential of crotonylation for targeted therapy.

**Methods:**

We systematically analyzed single-cell RNA-seq and bulk transcriptomic datasets from ovarian cancer patients. Cellular crotonylation activity was quantified using AUCell algorithm. Potential prognostic genes were identified through DEG analysis and Weighted gene correlation network analysis (WGCNA), and the associated molecular mechanisms were elucidated via Gene set enrichment analysis (GSEA). An ovarian cancer prognosis model were constructed by integrating machine learning algorithms. Immune microenvironment features were assessed using CIBERSORT, ESTIMATE and TIDE algorithms, with drug sensitivity predicted via genomics of drug sensitivity in cancer.

**Results:**

The ovarian cancer microenvironment is characterized by abundant immune cell infiltration, with significant differences in crotonylation levels among 7 cell subtypes. We identified 451 key crotonylation-related genes. The crotonylation risk score (RS) model demonstrated robust prognostic performance. High-RS groups showed immunosuppressive characteristics: decreased follicular helper T cells and activated NK cells, concomitant with M2 macrophage enrichment. Elevated RS was associated with increased stromal activation, as indicated by a higher ESTIMATE score, and enhanced immune evasion potential, reflected by an elevated TIDE score. Notably, high-RS patients exhibited upregulated PDL1 and CD40, suggesting increased immunotherapy susceptibility. Pharmacogenomic analysis identified vinblastine with differential sensitivity, providing actionable targets for RS-stratified therapy.

**Conclusion:**

We elucidated the significant impact of crotonylation on the ovarian cancer microenvironment and prognosis. We developed and validated a novel prognostic model for ovarian cancer that can serve as a tool for predicting patient outcomes and characterizing the immune microenvironment. These findings enhance our understanding of the role of crotonylation in ovarian cancer and establish a robust framework for developing therapeutic strategies targeting crotonylation.

## Introduction

Ovarian cancer (OV) is one of the most lethal malignant tumors in the female reproductive system, with approximately 70% of patients being diagnosed at an advanced stage (1, 2). Although surgery combined with platinum-based chemotherapy can temporarily alleviate this condition, widespread metastasis, recurrence, and drug resistance have resulted in a 5-year survival rate that has long remained below 40% (3, 4). In recent years, although precision medicine based on genetic testing (such as the use of PARP inhibitors) has led to treatment advances, there are still no widely applicable prognostic markers or effective intervention targets (2, 5). Therefore, identifying effective prognostic markers and therapeutic targets, as well as elucidating the key molecular mechanisms driving the initiation and progression of ovarian cancer, have become critical avenues to address the current clinical challenges.

In recent years, a growing body of research has demonstrated that epigenetic regulation is intricately linked to tumor microenvironment (TME) remodeling, potentially serving as a pivotal factor in the development and progression of ovarian cancer (6, 7). Protein crotonylation is a recently discovered form of acylation that was first identified in 2011 (8). Crotonylation is a modification that transfers a crotonyl group to lysine residues using crotonyl-CoA as the substrate; it is evolutionarily conserved and typically associated with actively transcribed chromatin regions, playing a crucial role in regulating gene expression (1, 8). Studies have demonstrated that crotonylation is closely associated with the proliferation, metastasis, and immune evasion of various tumors, including liver cancer and breast cancer (9–11). LINC00922 interacts with the protein sirtuin 3 (SIRT3) and inhibits its binding to the promoter region of ETS1, leading to an increase in H3K27 crotonylation (H3K27cr) levels in this region and the subsequent activation of ETS1 transcription (12). Hypoxia-induced decreases in PGK1 crotonylation promotes tumorigenesis by coordinating glycolysis with the TCA cycle (10). However, the expression patterns, prognostic value, and underlying mechanisms of crotonylation in ovarian cancer have not been systematically elucidated.

Despite efforts to identify prognostic genes for ovarian cancer and construct predictive models using transcriptome data, existing studies have several limitations. The adoption of a single-omics approach has restricted the integration of single-cell data, thereby hindering a comprehensive analysis of heterogeneous expression patterns of specific markers within distinct cell subpopulations in the TME. Consequently, the practical clinical utility of these models remains suboptimal. The sensitivity and specificity of existing markers, such as CA125 and HE4, are limited, highlighting the urgent need for multidimensional molecular signatures to enhance prognostic models. This study investigated the prognostic value and potential role of crotonylation in ovarian cancer.

In this study, we integrated transcriptomic and single-cell data to assess crotonylation levels across different cell subtypes. An ovarian cancer prognosis model were constructed by integrating machine learning algorithms, and the associated molecular mechanisms were elucidated via Gene set enrichment analysis (GSEA). Immune microenvironment features were assessed using CIBERSORT, ESTIMATE and TIDE algorithms, with drug sensitivity predicted via genomics of drug sensitivity in cancer.

## Methods and materials

### The research workflow of this study

As illustrated in **Supplementary Figure 1**, In scRNA-seq dataset, UMAP clustering revealed 20 cellular clusters. Manual annotation based on cell-type marker genes identified seven distinct cell populations. The “FindAllMarkers” function was employed to calculate differentially expressed genes between different cellular clusters. Using the AddModuleScore function, CRG scores were computed across these seven cell types. Additionally, the PercentageFeatureSet function was utilized to quantify CRG expression proportions within each cell type. Comparative analysis of CRG scores and expression was subsequently conducted across different sample types and cellular populations. Concurrently, we utilized the AUCell_calcAUC function from the R package AUCell to conduct AUCell scoring in the ovarian cancer samples of the scRNA-seq datasets. Using the AUCell_exploreThresholds function, the 7 different cell types in the OV samples were respectively divided into high/low AUC groups. The “FindMarkers” function subsequently identified differential expression genes between these AUC subgroups across cellular clusters, which were designated as DEG1 for subsequent investigation.

For bulk RNA-seq analysis, the GSVA package with ssGSEA algorithm was implemented to calculate CRG scores. Additionally, the Wilcoxon test was employed to evaluate the differences in CRG score across different samples. Differential expression analysis between ovarian cancer and normal samples in RNA-seq datasets was performed via the limma package, with the resultant genes defined as DEG2.

Co-expression module screening through WGCNA analysis in the RNA-seq datasets identified four significant modules. Pearson correlation analysis was performed to evaluate the correlation between modules and CRG score, and module eigengenes (MEGs) were identified based on these results. The intersection of DEG1, DEG2, and MEGs was ultimately established as key genes for this study.

Through integrative analysis, we identified key crotonylation-related genes and elucidated their underlying molecular mechanisms. Subsequently, we identified prognosis-associated genes and developed a novel prognostic signature, i.e. “risk score (RS),” using an ensemble machine learning algorithm. We investigated the biological mechanisms distinguishing high- and low-RS groups, as well as the relationships between RS and immune-related features, drug sensitivity, and highly variable gene (HGV) scores within the TME. Finally, we validated the expression patterns of the identified prognostic genes.

### Data acquisition and screening

scRNA-seq data from the OV dataset GSE184880 (13) were downloaded from the NCBI Gene Expression Omnibus (GEO) database (https://www.ncbi.nlm.nih.gov/geo/); this dataset comprised five normal samples and seven tumor samples. scRNA-seq datasets were normalized using the log-normalization method implemented in the NormalizeData function of the Seurat package (v4). The scRNA-seq data were processed and formatted as a Seurat object using the R package Seurat (https://satijalab.org/seurat/, version 4.4.0) (14), and the proportion of mitochondrial genes in each cell was calculated using the “PercentageFeatureSet” function in the Seurat package. Generally, and excessively high proportion of mitochondrial genes in a cell may indicate that the cell is undergoing apoptosis or has been lysed. Therefore, we excluded cells whose mitochondrial gene content exceeding 20%. Since low-quality cells or empty droplets typically have very few genes and doublets may exhibit an abnormally high number of genes, we also filtered out cells with fewer than 200 genes or more than 6,000 genes. Additionally, we removed cells with fewer than 100 detected genes or where fewer than 3 cells contained a given gene. After these quality control steps, a total of 47,897 cells were retained for subsequent single-cell analysis. The RNA-seq data for TCGA-OV, normalized to transcripts per kilobase per million mapped reads (TPM), were downloaded from the official The Cancer Genome Atlas (TCGA) portal (https://portal.gdc.cancer.gov/). The TPM values were transformed using log2(TPM + 1) transformation. After excluding samples lacking essential clinical information (FIGO stage, tumor grade, age, and follow-up data), a total of 414 OV samples remained in the TCGA-OV dataset. Since the TCGA database does not include normal ovarian tissue samples, we also obtained RNA-seq data for normal ovarian tissues from the Genotype Tissue Expression (GTEx) portal (https://www.gtexportal.org/home/); these data were also in TPM format and underwent the same log2(TPM + 1) transformation, resulting in a total of 193 control samples. The TCGA OV samples and the GTEx control samples were combined to form the training set for this study. We utilized the sva package (15) to remove batch effects arising from different database sources. We downloaded the OV-related datasets GSE140082 (16), GSE26193 (17), GSE26712 (18), and GSE63885 (19) from the GEO database (https://www.ncbi.nlm.nih.gov/geo/) using the GEOquery package (https://bioconductor.org/packages/release/bioc/html/GEOquery.html, version 2.70.0) (20). Among these datasets, GSE140082 was generated on the GPL14951 platform, GSE26193 and GSE63885 were generated on the GPL570 platform, and GSE26712 was generated on the GPL96 platform. We obtained 379 OV samples from the GSE140082 dataset, 107 OV samples from the GSE26193 dataset, 185 OV samples and 10 control samples from the GSE26712 dataset, and 75 OV samples from the GSE63885 dataset. These samples were included in this study as external validation datasets. Eighteen crotonylation-related genes (CRGs), including CREBBP, EP300, KAT8, KAT2A, KAT2B, KAT5, SIRT1, SIRT2, SIRT3, HDAC1, HDAC2, HDAC3, HDAC8, TAF1, MLLT3, YEATS2, KAT6A, and DPF2 were identified from previous literature (21).

### Cell subpopulation annotation

The Seurat package (version: 4.4.0) was used for cell normalization and regression based on the expression table according to the percentage of mitochondria to obtain the scaled data. Principal component analysis was constructed on the basis of the scaled data with the top 2,000 highly variable genes, and the top 20 principals were used for uniform manifold approximation and projection (UMAP) construction. Logarithmic normalization is applied for standardization. Canonical correlation analysis (CCA) in the Seurat package was applied for batch effect removal. The data were dimensionally reduced using the UMAP method with a resolution threshold=1, resulting in the identification of 20 cell clusters through clustering. We manually annotated these clusters and identified seven distinct cell types the basis of marker genes:

- B cells/plasma cells: CD79A and JCHAIN;
- Endothelial cells: PECAM1 and CLDN5;
- Epithelial cells: KRT18, EPCAM, CD24, and KRT19;
- Fibroblasts: DCN and OGN;
- Monocytes: CD14, C1QA, and CD4;
- SMC/myofibroblasts: ACTA2, MYH11, and TAGLN; and
- T cells: CD3D, CD3E, and CD8A.

The “FindAllMarkers” function was used to calculate differentially expressed genes between the various cell clusters, and the results were visualized using a heatmap.

### Calculation of CRG scores for single-cell data

On the basis of the aforementioned annotation results, we used the AddModuleScore function to calculate the CRG scores for the seven identified cell types. Additionally, the percentage feature set function was employed to determine the proportion of CRG expression in these cell types. Furthermore, we compared the differences in the CRG scores and CRG expression proportions across the different cell types among various sample types.

### AUCell scoring of cell populations

Using single-cell RNA sequencing data, AUCell (22) can identify cells with active gene sets. AUCell employs the “area under the curve” (AUC) method to determine whether a critical subset of an input gene set is enriched among the expressed genes in each cell. The distribution of AUC scores across all cells enables the exploration of relative feature expression. Since the scoring method is rank-based, AUCell is not influenced by gene expression units or standardization procedures. Additionally, because cells are evaluated individually, AUCell can be readily applied to larger datasets and expression matrices can be grouped as needed. We selected CRG and used the AUCell_calcAUC function in the R package AUCell (https://bioconductor.org/packages/release/bioc/html/AUCell.html, version 1.24.0) to calculate AUCell scores using single-cell data for OV samples. We subsequently set the random seed to 123456 and employed the AUCell_explore Thresholds function to classify the seven different cell types in the OV samples into high- and low-AUC groups. On the basis of the AUC grouping information described above, we used the “FindMarkers” function to calculate differentially expressed genes (DEGs) between the high- and low-AUC groups within each cell population. Additionally, genes with |log2FoldChange| > 0.25 and p value < 0.05 were selected as DEG1s for further analysis (23).

### Identification of key genes involved in crotonylation in ovarian cancer

The single-sample gene set enrichment analysis (ssGSEA) algorithm can quantify the relative abundance of each gene set in dataset samples. Therefore, we utilized the GSVA R package (https://bioconductor.org/packages/release/bioc/html/GSVA.html, version 1.50.1) (24) to calculate the crotonylation related genes (CRG) Score for each sample on the basis of the expression matrix of CRGs in the training set using the ssGSEA algorithm. Additionally, we employed the Wilcoxon rank-sum test to assess the differences in the CRG scores among different samples.

We then utilized the limma package (https://bioconductor.org/packages/release/bioc/html/limma.html, version 3.58.1) (25) to analyze DEGs between OV samples and normal samples in the training set. Genes with |logFC| > 1 and p value < 0.05 were selected as DEG2s for further analysis.

Weighted gene correlation network analysis (WGCNA) (26) aims to identify coexpressed gene modules, explore the relationships between gene networks and phenotypes, and study core genes within these networks. Candidate soft thresholds ranging from 1 to 20 were evaluated in the training set using the pick Soft Threshold function. The selection criteria were as follows: (1) the scale-free topological fit index (signed R²) should be ≥0.85 to ensure that the network exhibits scale-free characteristics; (2) select the minimum threshold should be selected while satisfying condition 1; and (3) the average connectivity should exhibit a steady downward trend to prevent overconnectivity. After a comprehensive evaluation, a soft threshold of 14 was selected as the optimal value. A scale-free network was subsequently constructed on the basis of this soft threshold, followed by the generation of the topological overlap matrix and hierarchical clustering. Gene modules were identified using dynamic tree cutting, with a minimum module size of 50 genes, and eigengenes were calculated for each module. The correlation between modules was assessed using their eigengenes, and hierarchical clustering was performed. Modules with correlations greater than 0.5 were merged, resulting in a final set of four modules. Pearson correlation analysis was then conducted to evaluate the relationships between modules and CRG scores, and module genes (MEGs) were subsequently screened out.

Finally, the intersection of DEG1s, DEG2s, and MEGs was determined to obtain the overlapping genes, which were identified as the key genes for this study.

### GO and KEGG enrichment analysis

To understand the functional enrichment and pathway enrichment of key genes, we conducted enrichment analysis. Gene Ontology (GO) analysis (27) is a widely used method for conducting large-scale functional enrichment studies, encompassing biological process (BP), molecular function (MF), and cellular component (CC) terms. The Kyoto Encyclopedia of Genes and Genomes (KEGG) (28) is a widely used database that stores information on genomes, biological pathways, diseases, and drugs. GO annotation analysis and KEGG pathway enrichment analysis of key genes were conducted using the R package clusterProfiler (29), with an FDR cutoff value of < 0.05 considered statistically significant.

### Construction of an OV prognostic model by integrating machine learning algorithms

First, we conducted univariate Cox regression analysis of the key genes identified in both the training set and the validation set to obtain genes with p < 0.1 and consistent hazard ratios across at least three datasets simultaneously (30). On the basis of the genes screened by univariate Cox regression analysis, an integrated computational framework was employed to identify potential prognostic genes and construct OV prognostic models. Initial gene signatures were constructed in TCGA-OV using ten machine learning algorithms, including random survival forest (RSF), elastic net (Enet), lasso, ridge, stepwise cox, CoxBoost, Cox partial least squares regression (plsRcox), supervised principal components (SuperPC), generalized boosted regression modeling (GBM), and survival support vector machine (survival-svm) (30, 31). Several of these algorithms possess feature selection capabilities, such as lasso, stepwise Cox, CoxBoost, and RSF. We integrated 10 algorithms to generate a model. To mitigate potential confounding effects from stochastic variation, a 10-fold cross-validation was implemented to ensure model robustness. On the basis of 10-fold cross-validation, 101 algorithmic combinations for the key genes identified were fitted to predict the survival outcomes of ovarian cancer patients. These 101 models were subsequently validated using four additional GEO datasets. To address batch effects across GEO datasets, batch correction was conducted utilizing the ComBat function from the sva package.

For each model, the c-index was calculated across all datasets, and the model with the highest average c-index was selected as the optimal model. The optimal model was used to construct an OV prognostic model, and the genes included in this model were identified as OV prognostic genes. The risk score (RS) for each patient was then calculated. The R package timeROC (https://cran.r-project.org/web/packages/timeROC/, version 0.4) (32) was used to validate the model through ROC curve analysis. The optimal cutoff point of the RS was determined using the surv_cutpoint function in the survminer package, on the basis of the survival time and survival status of OV patients (33). Patients were then divided into high- and low-RS groups according to this optimal cutoff value. Kaplan-Meier analysis was conducted using the “survival” (https://cran.r-project.org/web/packages/survival/index.html, versions 3.4-8) and “survminer” packages to investigate the correlation between patient survival time and RS.

On the basis of the results of the analysis of the OV prognosis model, we utilized the R package rms (https://cran.r-project.org/web/packages/rms/, version 6.8-0) to construct a nomogram (34). A nomogram is a graphical tool that represents the functional relationships among multiple independent variables using a series of nonintersecting line segments on a Cartesian coordinate plane; it is derived from multivariate regression analysis and employs specific scales to score each variable within the model. The total score is then calculated to predict the probability of an event occurring. Additionally, calibration curves and ROC curves at 1, 2, 3, 4 and 5 years were plotted to evaluate the predictive performance of the nomogram.

### High- and low-RS groups and immune-related characteristics

Using CIBERSORT (https://cibersort.stanford.edu/) (35), we calculated the proportions of 22 immune cell types on the basis of the TCGA OV tumor sample expression data. CIBERSORT is a tool that deconvolutes the expression matrix of immune cell subtypes using linear support vector regression. This method relies on a known reference set, which provides gene expression signatures for 22 immune cell subtypes to estimate immune cell infiltration.

The degree of infiltration of immune and stromal cells in the tumor microenvironment (TME) significantly impacts patient prognosis. To elucidate the prognostic implications of genes regulating immune and stromal cell activity, we employed the ESTIMATE algorithm (https://bioinformatics.mdanderson.org/estimate/), which uses transcriptional profiling of cancer samples to quantitatively assess the heterogeneity of cellular infiltration within the TME. We evaluated the immune activity of OV tumor samples using the expression profile matrix data via the R package “ESTIMATE” (36). With the ESTIMATE algorithm, we calculated the ESTIMATEScore, ImmuneScore, StromalScore, and TumorPurity to quantify the immune and stromal components within the samples.

On the basis of the RNA-seq data from the Tumor Immune Phenotype (TIP, http://biocc.hrbmu.edu.cn/TIP/) database, we analyzed and visualized the anticancer immune status of each sample in the high- and low-RS groups, as well as the proportions of tumor-infiltrating immune cells across the seven stages of the cancer immune cycle (37).

### GSEA enrichment analysis

To delineate biological pathway disparities across comparative cohorts, we conducted systematic gene set enrichment analysis (GSEA) (38) using transcriptomic datasets from ovarian cancer patients. GSEA is a computational method that can be used to determine whether a predefined set of genes shows statistically significant differences between two biological states and is commonly used to estimate changes in pathways and biological processes within expression datasets. Using the R package clusterProfiler, we selected GO(including biological process, cellular component, and molecular function) and KEGG pathway for the GSEA of genes that were differentially expressed genes between high- and low-RS OV groups. A false discovery rate (FDR) < 0.05 was considered to indicate significant enrichment.

### Drug sensitivity analysis of high- and low-RS groups

To investigate the sensitivity of ovarian cancer to common chemotherapy drugs, we utilized the Genomics of Drug Sensitivity in Cancer (GDSC, https://www.cancerrxgene.org/) database (39) to estimate the sensitivity of each patient to ovarian cancer chemotherapy drugs. We used the R package pRRophetic to calculate the half-maximal inhibitory concentration (IC50) (40). The Wilcoxon test was used to compare drug sensitivity differences among different RS groups. Additionally, we predicted responses to immunotherapy and immune escape effects using the Tumor Immune Dysfunction and Exclusion (TIDE) algorithm (41) (http://tide.dfci.harvard.edu).

### Identification of highly variable genes and calculation of highly variable gene scores

The FindAllMarkers function in the Seurat package was used to identify the top 100 highly variable genes (HVGs) for each cell population in OV samples. ssGSEA can be used to quantify the relative abundance of gene sets within individual samples. Therefore, we subsequently employed the R package GSVA (https://bioconductor.org/packages/release/bioc/html/GSVA.html, version 1.50.1) (24) to calculate the highly variable gene scores (HVGs) for each cell type in each OV sample from the TCGA dataset. This was achieved by applying the ssGSEA algorithm to the expression profiles of HVGs for each cell type in each sample.

### Immunohistochemical analysis based on the HPA database

We compared the immunohistochemical (IHC) expression patterns of prognostic genes between normal tissues and OV tumor tissues using the Human Protein Atlas (HPA) database (https://www.proteinatlas.org/) (42).

### Tissue specimens

We collected 16 OV tissues and 7 benign ovarian tumor tissues from Shanghai First Maternity and Infant Hospital. The OV patients were not received any preoperative radiation, chemotherapy, or other anticancer therapies before surgery. This study was approved by the Ethics Committee of Shanghai First Maternity and Infant Hospital. (No: KS25292). All subjects involved in this study signed informed consent documents prior to the operation.

### Real-time quantitative PCR

Total RNA from clinical tissue samples from patients with benign ovarian tumor and ovarian cancer was extracted by Trizol Reagent (Invitrogen, CA, USA), and cDNA synthesis was performed by using a PrimeScriptTM RT Master Mix Kit (TaKaRa BIO, Japan) according to the manufacturer’s protocol. The mRNA level was detected by using a Genious 2× SYBR Green Fast qPCR Mix (Low ROX Premixed) (Abclonal Bio, Wuhan, China) and an QuantaStudio™ -5 System(Thermo Fisher Scientific, MA, USA). The relative gene expression levels were calculated using the 2^−ΔΔCt^ method, and normalized by β-actin. All experiments were carried out in triplicate. The PCR primers were designed and synthesized by Sangon Biotech (Shanghai, China).

### Statistical analysis

All data calculations and statistical analyses were conducted using R programming (version 4.3.3). For comparisons of continuous variables between two groups, the statistical significance of normally distributed variables was assessed using the independent Student’s t-test, whereas differences in nonnormally distributed variables were analyzed using the Mann–Whitney U test (also known as the Wilcoxon rank sum test). Differences in categorical variables were evaluated using the chi-square test. The ggpubr R package was used to compare differences between two groups of data, and the survival package in R was used for survival analysis. Kaplan–Meier survival curves were generated to visualize survival differences, and the log-rank test was applied to assess the significance of survival time differences between patient groups. The survminer R package was utilized to visualize results. Unless otherwise specified, Spearman correlation analysis was performed to calculate the correlation coefficients between different molecules. All statistical p values in this study were two-sided, with p < 0.05 considered statistically significant.

## Results

### Single-cell heterogeneity in the ovarian cancer microenvironment

**Figure 1:**
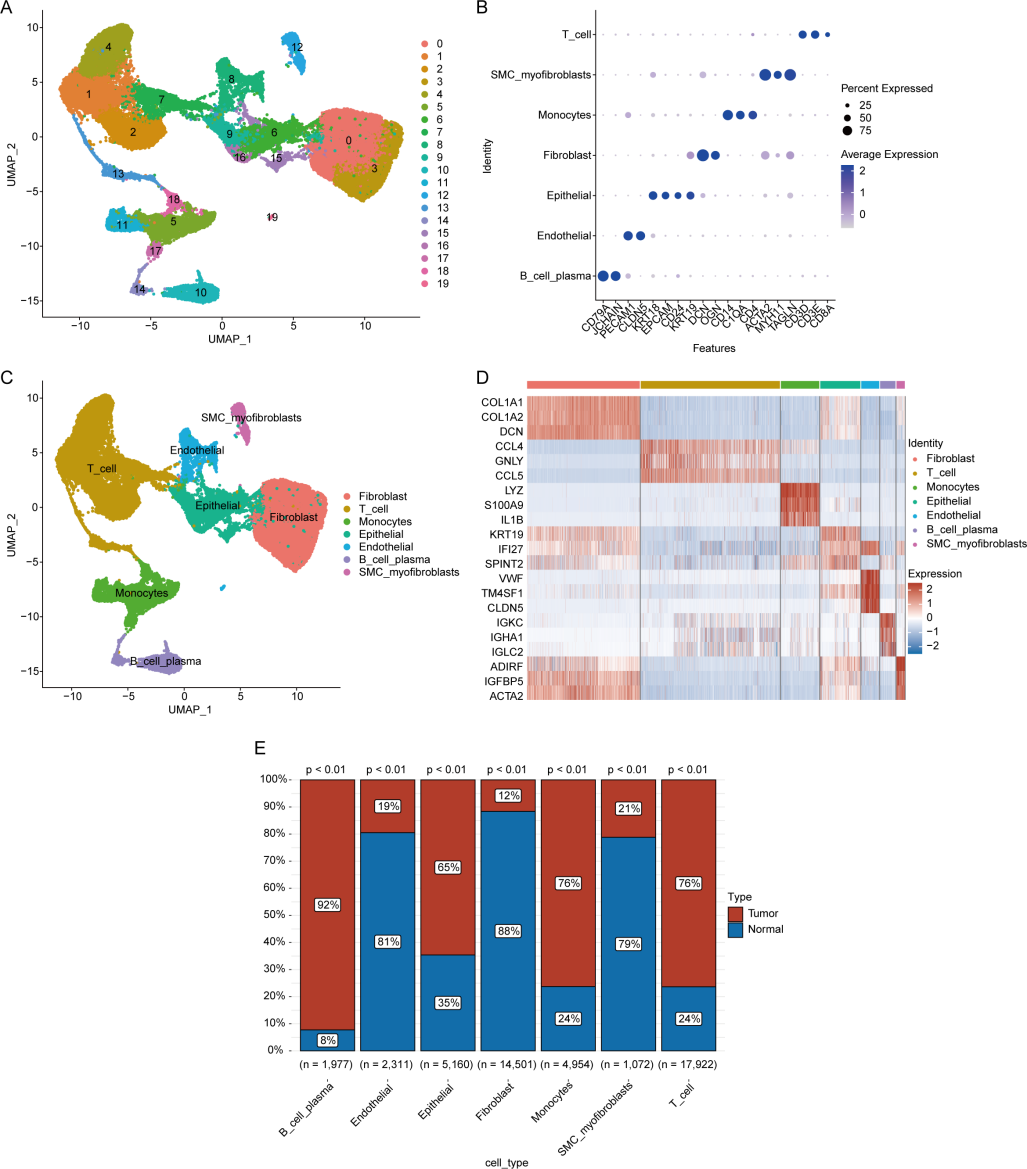
Single-cell analysis of the ovarian cancer microenvironment. **(A)** Clustering results for single-cell data. **(B)** Bubble plot depicting the expression of marker genes across different cell clusters. **(C)** Annotation results for identified cell clusters. **(D)** Heatmap showing differential gene expression in the single-cell transcriptome (displaying the top 20 genes; red indicates upregulation, and blue indicates downregulation). **(E)** Proportion of cell types in different samples.(Chi-squared test, without applying multiple testing corrections).

Twenty clusters were identified in the OV single-cell dataset through UMAP clustering, as detailed in the Methods section (**Figure 1A**). Manual annotation revealed seven distinct cell types within these clusters: B cells and plasma cells, endothelial cells, epithelial cells, fibroblasts, monocytes, SMC myofibroblasts, and T cells (**Figure 1C**). We analyzed the expression patterns of marker genes across single-cell subpopulations within different clusters and visualized the results in a bubble plot (**Figure 1B**). Additionally, through differential expression analysis, we identified differentially expressed genes (DEGs) in various cell types and presented the expression profiles of the top 20 DEGs for each cell type via a heatmap (**Figure 1D**). Finally, we compared the proportions of each cell type between normal and tumor tissues (**Figure 1E**). The results demonstrated that the proportions of B cells, plasma cells, epithelial cells, monocytes, and T cells were significantly greater in the ovarian cancer samples than in control samples. In contrast, endothelial cells, fibroblasts, and smooth muscle cell (SMC) myofibroblasts were more abundant in normal tissues. These findings suggest that immune cell infiltration is prominent in the ovarian cancer microenvironment. However, mechanisms remain poorly understood. Further exploration of this process could facilitate the development of therapeutic strategies targeting the immune microenvironment.

### Crotonylation in the ovarian cancer microenvironment

**Figure 2:**
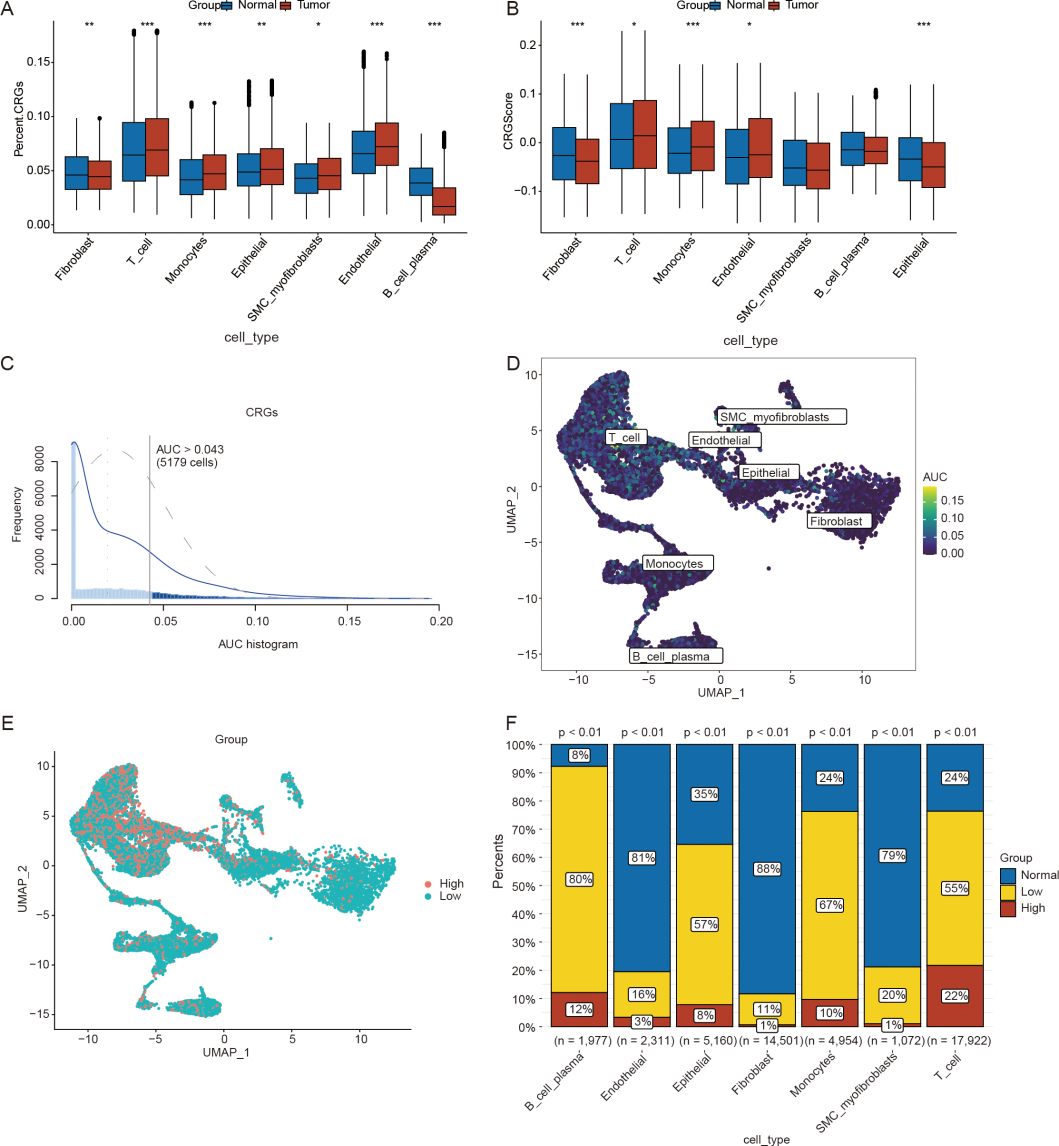
Calculation of the scores for crotonylation-related genes(CRGs) using single-cell data. **(A)** Differences in the expression levels of CRGs across different cell types. **(B)** Differences in CRG scores among various cell types. **(C)** AUCell scores calculated on the basis of CRGs in ovarian cancer(OV) samples from the single-cell dataset. **(D)** UMAP plot of AUCell scores derived from CRGs in ovarian cancer(OV) samples from the single-cell dataset. **(E)** UMAP plot illustrating the distribution of the high- and low-AUCell score groups. **(F)** Differences in the proportions of cell clusters between the high- and low-AUCell score groups. (*p < 0.05; **p < 0.01; ***p < 0.001) (Wilcoxon rank sum test and Chi-squared test, without applying multiple testing corrections).

To investigate the potential role of crotonylation in the ovarian cancer microenvironment, we systematically profiled the expression of crotonylation-related genes (CRGs) across cellular subtypes using single-cell transcriptomics. The percentageFeatureSet function was used to calculate the expression levels of CRGs in the seven aforementioned cell types. The results demonstrated that, in ovarian cancer samples, the expression levels of CRGs were significantly elevated in endothelial cells, epithelial cells, monocytes, SMC myofibroblasts, and T cells compared to the control group. In contrast, the expression levels of CRGs in B cells were markedly reduced relative to the control group (**Figure 2A**). The AddModuleScore function in the Seurat package was subsequently utilized to compute the CRG scores across different cell types. The analysis revealed significant differences in CRG scores among endothelial cells, epithelial cells, fibroblasts, monocytes, and T cells across different sample types (**Figure 2B**).

To verify the activity levels of CRGs across different cell types, we utilized the AUCell package to calculate the CRG activity in each cell type within the OV samples (**Figures 2C, D**). On the basis of the threshold of 0.043 provided by the AUCell_exploreThresholds function, cells from the OV samples were categorized into high- and low-AUC groups (**Figure 2E**). We subsequently employed the chi-square test to compare the distributions of different cell types between the high- and low-AUC groups. The results indicated that the activity of CRGs was significantly greater in B and plasma cells, monocytes, epithelial cells, and T cells than in the other cell types (p < 0.05) (**Figure 2F**). Crotonylation results in abnormal cell type-specific activation in ovarian cancer, which can serve not only as a biomarker for molecular stratification but also as a potential driver of disease progression by regulating the heterogeneity and plasticity of the TME. Consequently, crotonylation may emerge as a valuable tool for predicting the prognosis of patient with ovarian cancer.

### Identification of key genes and their potential biological mechanisms in ovarian cancer progression based on the basis of CRGs

To delineate the prognostic utility of crotonylation in ovarian cancer and elucidate the underlying mechanisms, we employed a multi-omics integrative approach to systematically identify key genes associated with CRGs. First, we identified differentially expressed genes (DEGs) between high- and low-CRG scores groups for each cell population and selected DEGs on the basis of |log2FoldChange| > 0.25 and p < 0.05. There were 197 DEGs in B cells and plasma cells (**Supplementary Figure 2A**), 451 DEGs in endothelial cells (**Supplementary Figure 2B**), 420 DEGs in epithelial cells (**Supplementary Figure 2C**), 562 DEGs in fibroblasts (**Supplementary Figure 2D**), 610 DEGs in monocytes (**Supplementary Figure 2E**), 1,148 DEGs in SMC myofibroblasts (**Supplementary Figure 2F**), and 350 DEGs were identified in T cells (**Supplementary Figure 2G**). The results of the differential expression analysis are summarized in **Supplementary File 1**. We merged the differentially expressed genes (DEGs) between the high and low-CRG score groups across various cell populations, resulting in a total of 2,618 genes. These genes were designated as DEG1s for this study (**Supplementary File 2**).

**Figure 3:**
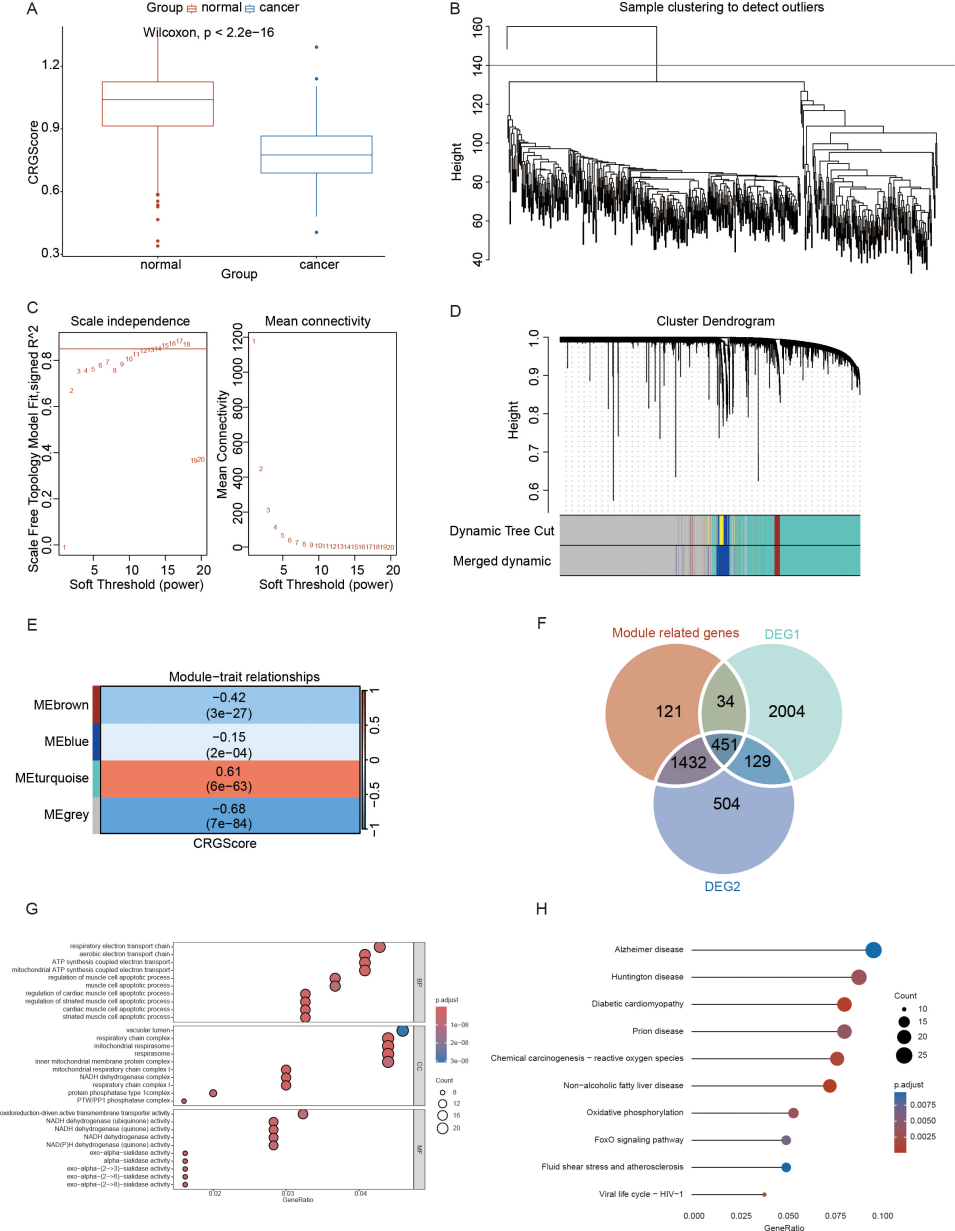
Identification of key genes and their potential biological mechanisms in ovarian cancer progression on the basis of crotonylation related genes(CRGs). **(A)** Distribution of CRG scores across different sample types in the training set. **(B)** Cluster tree diagram after removing outlier samples using a cut height threshold. **(C)** Determination of the optimal soft-threshold power. **(D)** Analysis of the aggregation process of module genes. **(E)** Correlation heatmap between modules and CRG scores. **(F)** Intersection of genes from the Module related genes, DEG1s, and DEG2s. **(G)** Gene ontology (GO) enrichment analysis. **(H)** The Kyoto encyclopedia of genes(KEGG) enrichment analysis. (Wilcoxon rank sum test, without applying multiple testing corrections).

To compare the differences in gene expression patterns between OV tumor samples and normal samples, we conducted differential expression analysis using the limma package. The analysis revealed 1,074 upregulated genes and 1,442 downregulated genes (**Supplementary Figure 3**). These differentially expressed genes (DEGs) between ovarian cancer samples and normal samples were designated as DEG2s. The complete list of genes is provided in **Supplementary File 3**. In accordance with the method described, the CRG Score for each sample in the training set was calculated on the basis of the CRG expression matrix of each sample through the ssGSEA algorithm. The differences in CRG Scores between OV tumor samples and normal samples were compared. The analysis results revealed that the CRG Score of the tumor samples was significantly lower than that of normal samples (**Figure 3A**).

We subsequently conducted WGCNA using the training dataset to identify coexpression modules. During the WGCNA analysis, we excluded one outlier sample by setting a cutoff height threshold (**Figure 3B**). Using a scatter plot, we determined that a soft threshold of 14 was optimal and proceeded with subsequent analyses (**Figure 3C**). The module merge and cut height was subsequently set to 0.5. Modules with merged and cut heights lower than 0.5 were merged and sheared. The genes in the training set were clustered into four modules (MEbrown, MEblue, MEturquoise, and MEgrey) (**Figure 3D**). Pearson correlation analysis was used to determined the correlation between each module and the CRG scores (**Figure 3E**) and selected the module with the highest correlation, namely, the MEturquoise module was selected for further analysis. The MEturquoise module contained 2,038 genes. The list of genes is provided in **Supplementary File 4**. We intersected the genes within the MEturquoise module, DEG1s, and DEG2s, resulting in 451 key genes for this study (**Figure 3F**). A list of these genes is provided in **Supplementary File 5**.

To explore the potential biological mechanisms underlying the key genes, we conducted GO and KEGG enrichment analyses of the 451 identified key genes. The results of these analyses are summarized in **Supplementary File 6** (GO) and **Supplementary File 7** (KEGG). GO analysis revealed that the key genes were associated with biological processes such as the regulation of cardiac muscle cell apoptosis, the aerobic electron transport chain, and the regulation of striated muscle cell apoptosis. These genes were also linked to cellular components such as respiratory chain complexes, mitochondrial respirasomes, and respirasomes, as well as molecular functions such as NADH dehydrogenase (ubiquinone) activity, NADH dehydrogenase (quinone) activity, and exo-alpha-sialidase activity (**Figure 3G**). The KEGG analysis revealed that the key genes were involved in pathways related to nonalcoholic fatty liver disease, diabetic cardiomyopathy, and chemical carcinogenesis via reactive oxygen species (**Figure 3H**).

### Construction of a prognostic model of ovarian cancer on the basis of CRGs

**Figure 4:**
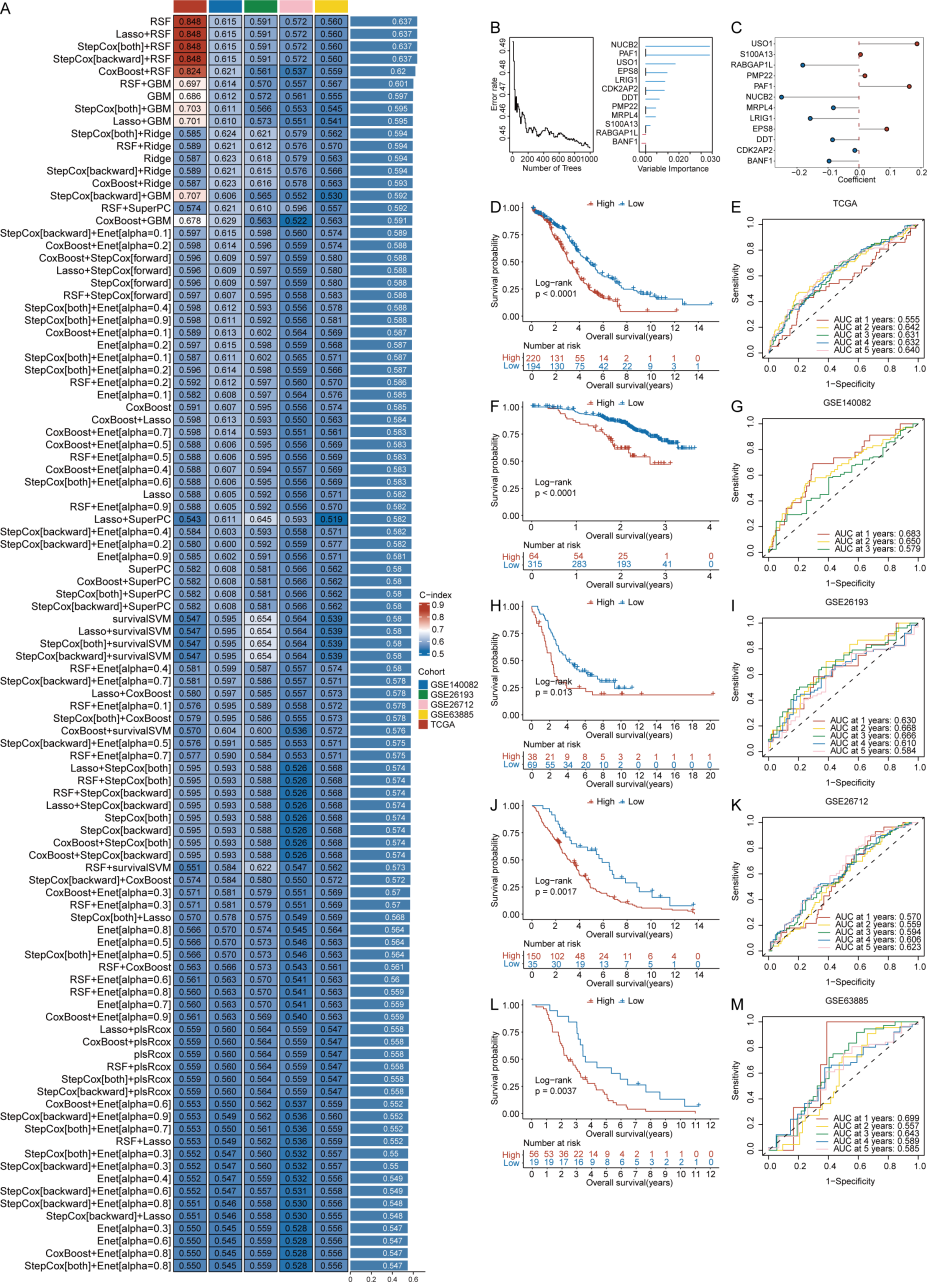
Construction of a prognostic model of ovarian cancer on the basis of crotonylation related genes(CRGs). **(A)** C-index values for 101 machine learning algorithm combinations across five cohorts. **(B)** Random Survival Forests(RSF) analysis results. **(C)** Risk regression coefficients for 12 genes. **(D)** Survival curves comparing high- and low-risk scores (RS) groups in the TCGA cohort. **(E)** ROC analysis for predicting 1, 2, 3, 4, and 5 year overall survival in the TCGA cohort. **(F)** Survival curves comparing high- and low-risk scores(RS) groups in the GSE140082 cohort. **(G)** ROC analysis for predicting 1, 2, and 3 year overall survival in the GSE140082 cohort. **(H)** Survival curves comparing high and low-risk scores(RS) groups in the GSE26193 cohort. **(I)** ROC analysis for predicting 1, 2, 3, 4, and 5 year overall survival in the GSE26193 cohort. **(J)** Survival curves comparing high and low-risk scores(RS) groups in the GSE26712 cohort. **(K)** ROC analysis for predicting 1, 2, 3, 4, and 5-year overall survival in the GSE26712 cohort. **(L)**. Survival curves comparing high and low-risk scores(RS) groups in the GSE63885 cohort. **(M)**. ROC analysis for predicting 1, 2, 3, 4, and 5 year overall survival in the GSE63885 cohort. (Log-rank test, without applying multiple testing corrections).

To investigate the prognostic value of DEGs associated with CRGs in ovarian cancer, we constructed a risk model to evaluate their impact on patient prognosis. On the basis of the 451 key DEGs identified, we performed univariate Cox regression analysis separately using the tumor samples of the training set and validation set, resulting in the identification of 13 genes (BANF1, CDK2AP2, CYBA, DDT, EPS8, LRIG1, MRPL4, NUCB2, PAF1, PMP22, RABGAP1L, S100A13 and USO1). We constructed prediction models using 101 algorithm combinations through 10-fold cross-validation using TCGA OV samples. Model evaluation focused primarily on their robustness across different validation cohorts. Consequently, we also assessed the performance of these models using four additional test cohorts and calculated the average c-index for each algorithm (**Figure 4A**). Ultimately, we selected the RSF model, which had the highest average c-index (0.637). On the basis of the RSF analysis of the 13 genes, 12 genes were retained as prognostic markers (**Figure 4B**).

A proportional hazards regression model was used to calculate the risk regression coefficients for the 12 genes (**Figure 4C**), and a formula for calculating the risk score (RS) was constructed. The calculation formula is as follows: RS = (-0.250 * NUCB2 expression) + (-0.014 * CDK2AP2 expression) + (0.162 * PAF1 expression) + (-0.157 * LRIG1 expression) + (-0.083 * MRPL4 expression) + (0.090 * EPS8 expression) + (0.187 * USO1 expression) + (-0.085 * DDT expression) + (0.019 * PMP22 expression) + (-0.181 * RABGAP1L expression) + (0.005 * S100A13 expression) + (-0.097 * BANF1 expression).

After the risk scores (RS) for ovarian cancer patients were obtained, the patients were grouped according to the cutoff value provided by the surv_cutpoint function from the survminer package in R. Survival analysis was conducted on basis of the group information, and survival curves were generated. The survival probability of the low-RS groups in the TCGA cohort was significantly greater than that of the high-RS groups (p < 0.0001) (**Figure 4D**). Similar results were observed for the GSE140082 cohort (p < 0.0001) (**Figure 4F**), the GSE26193 cohort (p = 0.013) (**Figure 4H**), the GSE26712 cohort (p = 0.0017) (**Figure 4J**), and the GSE63885 cohort (p = 0.0037) (**Figure 4L**).

We subsequently developed a prognostic model for performance evaluation. The results demonstrated that our model exhibited superior performance in the TCGA cohort. Specifically, the AUC values at 1, 2, 3, 4, and 5 years were 0.555, 0.642, 0.640, 0.631, and 0.632, respectively (**Figure 4E**). Notable performance was also observed in the validation cohorts. In the GSE140082 cohort, the time-dependent AUC values for 1, 2, and 3 years were 0.683, 0.650, and 0.579, respectively (**Figure 4G**). For the GSE26193 cohort, the AUC values at 1, 2, 3, 4, and 5 years were 0.630, 0.668, 0.666, 0.610, and 0.584, respectively (**Figure 4I**). In the GSE26712 cohort, the AUC values for 1, 2, 3, 4, and 5 years were 0.570, 0.559, 0.594, 0.606, and 0.623, respectively (**Figure 4K**). Finally, in the GSE63885 cohort, the AUC values for 1, 2, 3, 4, and 5 years were 0.699, 0.557, 0.643, 0.589, and 0.585, respectively (**Figure 4M**).

### Construction of a nomogram for ovarian cancer patients based on the RS

**Figure 5:**
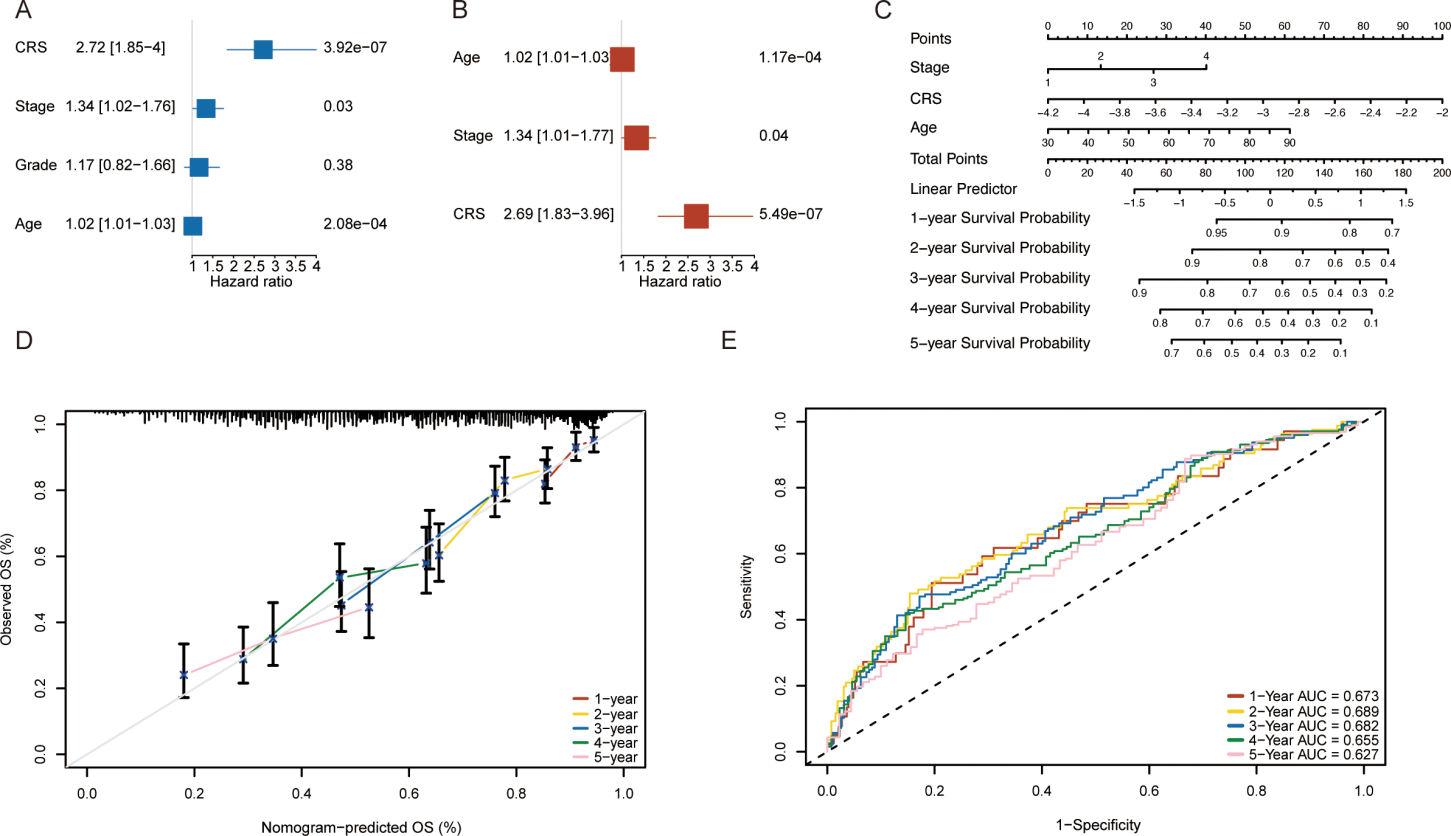
Construction of a nomogram on the basis of the risk scores(RS) for ovarian cancer patients in the TCGA database. **(A)** Univariate Cox analysis results. **(B)** Multivariate Cox analysis results. **(C)** Nomogram based on clinical characteristics. **(D)** Calibration curves for 1-, 2-, 3-, 4- and 5-year survival. **(E)** ROC analysis for predicting 1-, 2-, 3-, 4- and 5-year overall survival rates via the nomogram model.

To evaluate whether factors such as patient age, tumor stage, and the RS could be considered independent prognostic factors for OV patients, we constructed a nomogram for OV patients. We first performed univariate analysis and subsequently incorporated key clinical variables, including age and stage into multivariate risk regression analyses. Univariate risk regression analysis revealed that patient age (p < 0.001), tumor stage (p = 0.03), and RS (p < 0.001) were significantly associated with ovarian cancer prognosis (**Figure 5A**). Therefore, we selected factors with p values less than 0.05 from the univariate Cox analysis for inclusion in the multivariate risk regression analysis. The results indicated that patient age (p < 0.001), tumor stage (p = 0.04), and RS (p < 0.001) were independently associated with ovarian cancer prognosis and could serve as prognostic factors (**Figure 5B**). Finally, a nomogram was constructed on the basis of the clinical features of age, RS, and tumor stage (**Figure 5C**). We plotted calibration curves to evaluate the performance of the nomogram model. **Figure 5D** shows the comparison between the nomogram model and the ideal model at 1, 2, 3, 4, and 5 years. The results indicated that the nomogram model was most consistent with the ideal model at 1 year; the other time points also showed substantial consistency, suggesting the high accuracy of our model. Finally, we evaluated the overall performance of the nomogram model. The results demonstrated that the nomogram model exhibited good predictive performance, with time-dependent AUCs of 0.673, 0.689, 0.682, 0.655, and 0.627 at 1, 2, 3, 4, and 5 years, respectively (**Figure 5E**).

### Potential biological mechanisms and immunological characteristics of the RS

**Figure 6:**
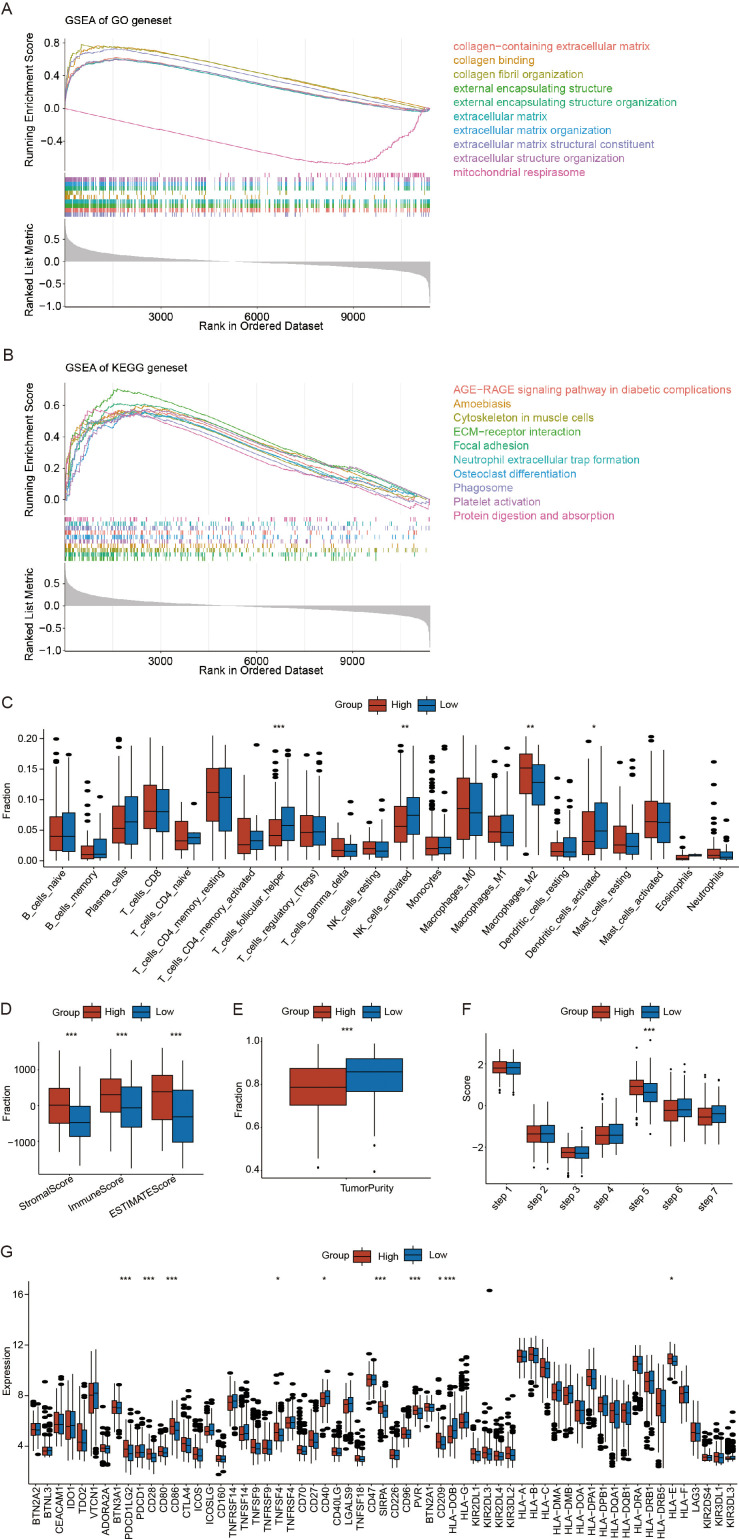
Potential biological mechanisms and immunological characteristics of the risk scores(RS). **(A)** Gene Set Enrichment Analysis (GSEA) enrichment analysis of Gene ontology (GO) related gene sets. **(B)** GSEA enrichment analysis of Kyoto encyclopedia of genes(KEGG) related gene sets. **(C)** Differences in immune cell infiltration between the high- and low-RS groups. **(D)** Differences in the StromalScore, ImmuneScore, and ESTIMATEScore between high- and low-RS groups. **(E)** Differences in tumor purity between the high- and low-RS groups. **(F)** Differences in the tumor immune cycle between the high- and low-RS groups. **(G)** Differences in immune checkpoint gene expression between the high- and low-RS groups. (*p < 0.05; **p < 0.01; ***p < 0.001) (Wilcoxon rank sum test, without applying multiple testing corrections).

To explore the potential biological mechanisms underlying the RS, we first used the limma package to conduct differential expression analysis between the high- and low-RS groups in the training set of OV samples. We obtained the fold changes in expressed for the differentially genes and ranked genes accordingly. We subsequently performed GSEA enrichment analysis using both GO-related and KEGG-related gene sets. GSEA enrichment analysis results are provided in **Supplementary File 8**. **Figure 6A** displays the top 10 enriched terms for the GO-related gene set, including extracellular matrix structural constituent, collagen-containing extracellular matrix, and external encapsulating structure. **Figure 6B** shows the top 10 enriched pathways for the KEGG-related gene set, including ECM-receptor interaction, focal adhesion, and cytoskeleton organization in muscle cells.

To investigate the association between the RS and immune microenvironment, we analyzed its relationship with infiltrating immune cell populations. Using the CIBERSORT algorithm, as described in the methods, we estimated the relative abundance of infiltrating immune and stromal cells. The results demonstrated significant associations between crotonylation risk scores and the infiltration levels of various immune cell populations, including T_cells_follicular_helper, NK_cells_activated, Macrophages_M2, and Dendritic_cells_activated(**Figure 6C**). High-RS groups showed immunosuppressive characteristics: decreased follicular helper T cells and activated NK cells, concomitant with M2 macrophage enrichment (p < 0.05) (**Figure 6C**). To further investigate the immune features associated with RS, we utilized the ESTIMATE algorithm to calculate the StromalScore, ImmuneScore, ESTIMATEScore, and TumorPurity. The results indicated that high-RS was associated with increased stromal activation, as indicated by a higher StromalScore and ESTIMATE score, and enhanced immune evasion potential, reflected by an elevated TIDE score (p < 0.05) (**Figure 6D**). However, the tumor purity was significantly greater in the low-RS groups than in the high-RS groups (p < 0.05) (**Figure 6E**). Furthermore, we analyzed the differences in the RS within the tumor immune cycle by tracking the tumor immune phenotype in ovarian cancer, and the results indicated that step 5 was significantly activated in the high-RS compared to the low-RS groups. (p < 0.05) (**Figure 6F**). Finally, we employed the Wilcoxon rank-sum test to compare the expression levels of immune checkpoint genes between the high- and low-RS groups. The results revealed that the expression of nine immune checkpoint genes was significantly different between these two groups, Notably, high-RS patients exhibited upregulated PDL1 and CD40 (p < 0.05) (**Figure 6G**). The above findings indicate that the high RS groups exhibits increased susceptibility to immunotherapy.

### Predicting the drug sensitivity of ovarian cancer patients using the RS

**Figure 7:**
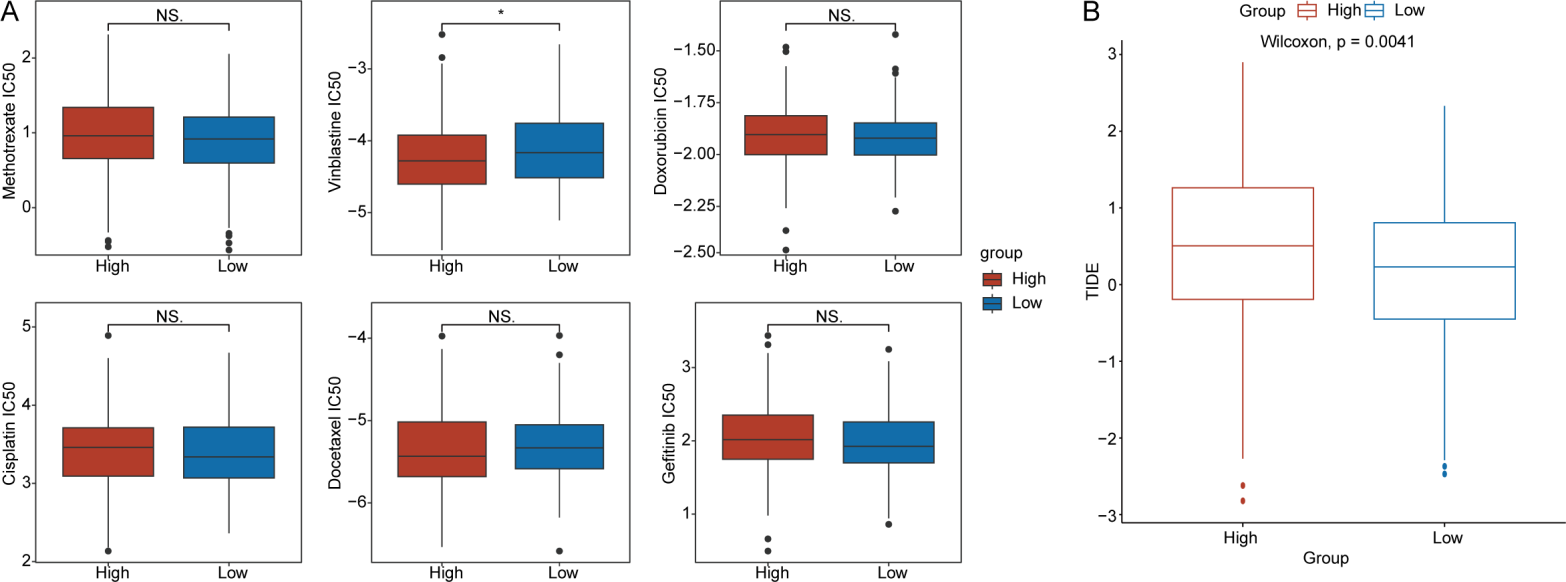
Drug sensitivity analysis. **(A)** Differences in the half-maximal inhibitory concentration(IC50) values for methotrexate, vinblastine, doxorubicin, cisplatin, docetaxel, and gefitinib between the high- and low-RS groups. **(B)** Distribution of tumor immune dysfunction and exclusion(TIDE) scores for the high- and low-RS groups. (*p < 0.05; ns, no significance).

In accordance with the methods described, we calculated the IC50 values of chemotherapy drugs, including methotrexate, vinblastine, doxorubicin, cisplatin, docetaxel, and gefitinib, in OV patients (**Figure 7A**). We found that the IC50 value of vinblastine was significantly greater in the low-RS groups than in the high-RS groups (p < 0.05) (**Figure 7A**). Additionally, we explored the relationship between the RS and the immune therapy predictor TIDE. The results indicated that TIDE scores were significantly higher in the high-RS groups than in the low-RS groups (p < 0.05) (**Figure 7B**).

### Analysis of highly variable genes between the high- and low-RS groups in the ovarian cancer microenvironment

To further validate the critical role of crotonylation within the ovarian cancer microenvironment, we examined the association between RS and highly variable gene (HGV) scores across different cell subtypes, as well as their influence on patient survival rates. First, as detailed in the Methods section, we utilized the FindAllMarkers function from the Seurat package to identify the top 100 HVGs for each cell population in the ovarian cancer samples (**Supplementary File 9**). Using the ssGSEA algorithm, we subsequently calculated HVG scores for each cell population in the training set. We then employed the Wilcoxon rank sum test to compare the distribution of HVG scores between the high- and low-RS groups. Additionally, we utilized the surv_cutpoint function in the survminer package’s, incorporating patient survival time and status as target variables, to determine the optimal cutoff values for the HVG scores for each cell population. Patients were stratified into high- and low-HVG score groups on the basis of these cutoff values. Then, Kaplan–Meier analysis was conducted to evaluate the association between patient survival time and the HVG score of each cell population.

**Figure 8:**
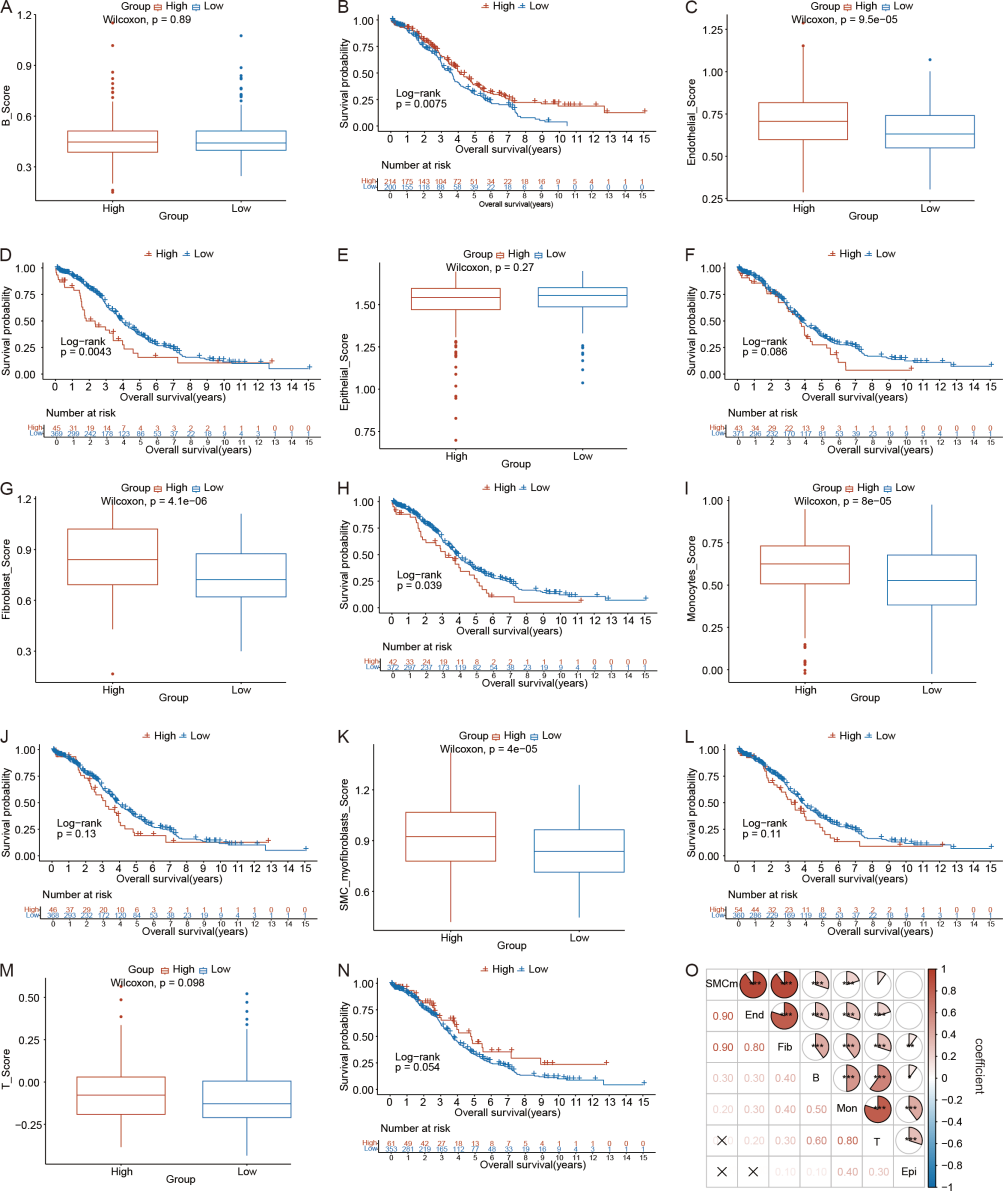
Analysis of highly variable genes(HVGs) in the ovarian cancer microenvironment between the high- and low-RS groups. **(A)** Distribution differences in the HVG scores for B cells and plasma cells between the high- and low-RS groups. **(B)** Survival analysis results for HVG scores for B cells and plasma cells. **(C)** Distribution differences in the HVG scores for endothelial cells between the high- and low-RS groups. **(D)** Survival analysis results for HVG scores for endothelial cells. **(E)** Distribution differences in HVG scores for epithelial cells between the high- and low-RS groups; **(F)** Survival analysis results for HVG scores for epithelial cells. **(G)** Distribution differences in HVG scores for fibroblasts between the high- and low-RS groups; **(H)** Survival analysis results for HVG scores for fibroblasts. **(I)** Distribution differences in the HVG scores for monocytes between the high- and low-RS groups. **(J)** Survival analysis results for HVG scores for monocytes. **(K)** Distribution differences in HVG scores for SMC myofibroblasts between the high- and low-RS groups. **(L)** Survival analysis results for HVG scores for SMC myofibroblasts. **(M)** Distribution differences in the HVG scores for T cells between the high- and low-RS groups. **(N)** Survival analysis results for HVG scores for T cells. **(O)** Heatmap of HVG score correlations (red indicates a positive correlation, blue indicates a negative correlation). (*p < 0.05; **p < 0.01; ***p < 0.001) (Wilcoxon rank sum test, without applying multiple testing corrections).

**Figure 9:**
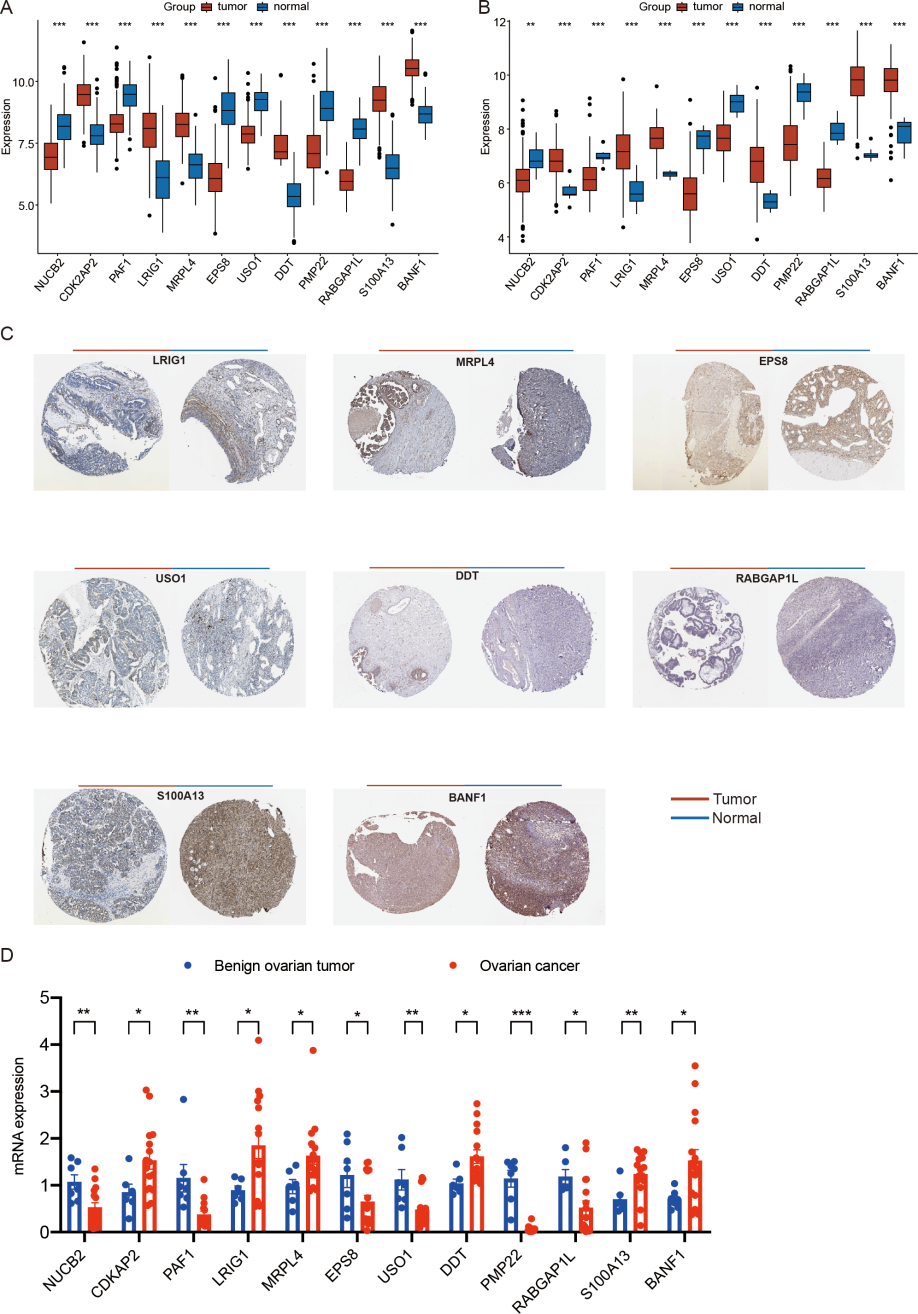
Expression analysis of ovarian cancer prognostic genes. **(A)** the expression of ovarian cancer prognostic genes among different types of samples in the training set. **(B)** the expression of ovarian cancer prognostic genes among different types of samples in the GSE26712 validation set. **(C)** Immunohistochemical analysis results for OV prognostic genes in the HPA database (BANF1, CDK2AP2, DDT, EPS8, LRIG1, MRPL4, NUCB2, PAF1, PMP22, RABGAP1L, S100A13, and USO1) **(D)** the mRNA expression of ovarian cancer prognostic genes among clinical tissue samples from patients with benign ovarian tumor and ovarian cancer by qRT-PCR.(*p < 0.05; **p < 0.01; ***p < 0.001). (Wilcoxon rank sum test, without applying multiple testing corrections).

The analysis revealed no significant difference in the HVG scores for B cells and plasma cells between the high- and low-RS groups (p = 0.89) (**Figure 8A**). However, patients with higher HVG scores for B cells and plasma cells exhibited a significantly greater survival probability compared to those with lower HVG scores (p = 0.0075) (**Figure 8B**). For endothelial cells, HVG scores were significantly higher in the high-RS groups than in the low-RS groups (p = 9.5 × 10^-5^) (**Figure 8C**), and patients with higher HVG scores for endothelial cells had a significantly lower survival probability than those with lower HVG scores (p = 0.0043) (**Figure 8D**). No significant difference was observed in the HVG scores for epithelial cells between the high- and low-RS groups (p = 0.27) (**Figure 8E**), nor was there a significant difference in survival probability between patients with high and low HVG scores for epithelial cells (p = 0.086) (**Figure 8F**). Fibroblasts showed significantly increased HVG scores in the high-RS groups compared to the low-RS groups (p = 4.1 × 10^-6^) (**Figure 8G**), and patients with higher HVG scores for fibroblasts had a significantly lower survival probability than those with lower HVG scores (p = 0.039) (**Figure 8H**). Monocytes demonstrated significantly increased HVG scores in the high-RS groups compared to the low-RS groups (p = 8 × 10^-5^) (**Figure 8I**), but no significant difference in survival probability was observed between patients with high and low HVG scores for monocytes (p = 0.13) (**Figure 8J**). SMC myofibroblasts also exhibited significantly increased HVG scores in the high-RS groups compared to the low-RS groups (p = 4 × 10^-5^) (**Figure 8K**), yet no significant difference in survival probability was noted between patients with high and low HVG scores for SMC myofibroblasts (p = 0.11) (**Figure 8L**). Finally, no significant difference was found in the HVG scores for T cells between the high- and low-RS groups (p = 0.098) (**Figure 9M**), nor was there a significant difference in survival probability between patients with high and low HVG scores for T cells (p = 0.054) (**Figure 8N**).

Finally, we analyzed the correlations among the HVG scores for the seven cell populations. The results demonstrated that the HVG scores for all seven cell types were positively correlated. Notably, the strongest positive correlations were observed between SMC myofibroblasts and endothelial cells (r = 0.90, p < 0.001) and between SMC myofibroblasts and fibroblasts (r = 0.90, p < 0.001) (**Figure 8O**).

### Expression and immunohistochemical analysis of prognosis-related genes in ovarian cancer

To further analyze the expression of prognosis-related genes in ovarian cancer, we compared the expression levels of the 12 identified prognostic genes (BANF1, CDK2AP2, DDT, EPS8, LRIG1, MRPL4, NUCB2, PAF1, PMP22, RABGAP1L, S100A13, and USO1) across different sample types. The analysis revealed that in the training set, the expression of BANF1, CDK2AP2, DDT, LRIG1, MRPL4, and S100A13 was significantly upregulated in tumor samples, and the expression of EPS8, NUCB2, PAF1, PMP22, RABGAP1L, and USO1 was higher in control samples (p < 0.05) (**Figure 9A**). Consistent with these findings, in the GSE26712 validation set, the expression of BANF1, CDK2AP2, DDT, LRIG1, MRPL4, and S100A13 was significantly upregulated in tumor samples, and the expression of EPS8, NUCB2, PAF1, PMP22, RABGAP1L, and USO1 was higher in control samples (p < 0.05) (**Figure 9B**).

We subsequently retrieved immunohistochemical results for the prognostic genes in ovarian cancer (OV) from the HPA database. The results revealed notable differences in the protein expression levels of these genes between OV tumor tissues and normal tissues (**Figure 9C**).

Furthermore, We collected clinical tissue samples from patients with benign ovarian tumor and ovarian cancer. we performed quantitative analysis of prognostic genes (NUCB2, CDK2AP2, PAF1, LRIG1, MRPL4, EPS8, USO1, DDT, PMP22, RABGAP1L, S100A13, BANF1) via qRT-PCR. The results showing high consistency with the findings derived from scRNA-seq and transcriptome datasets (p < 0.05) (**Figure 9D**).

### Correlation analysis between prognostic genes and immune cells

**Figure 10:**
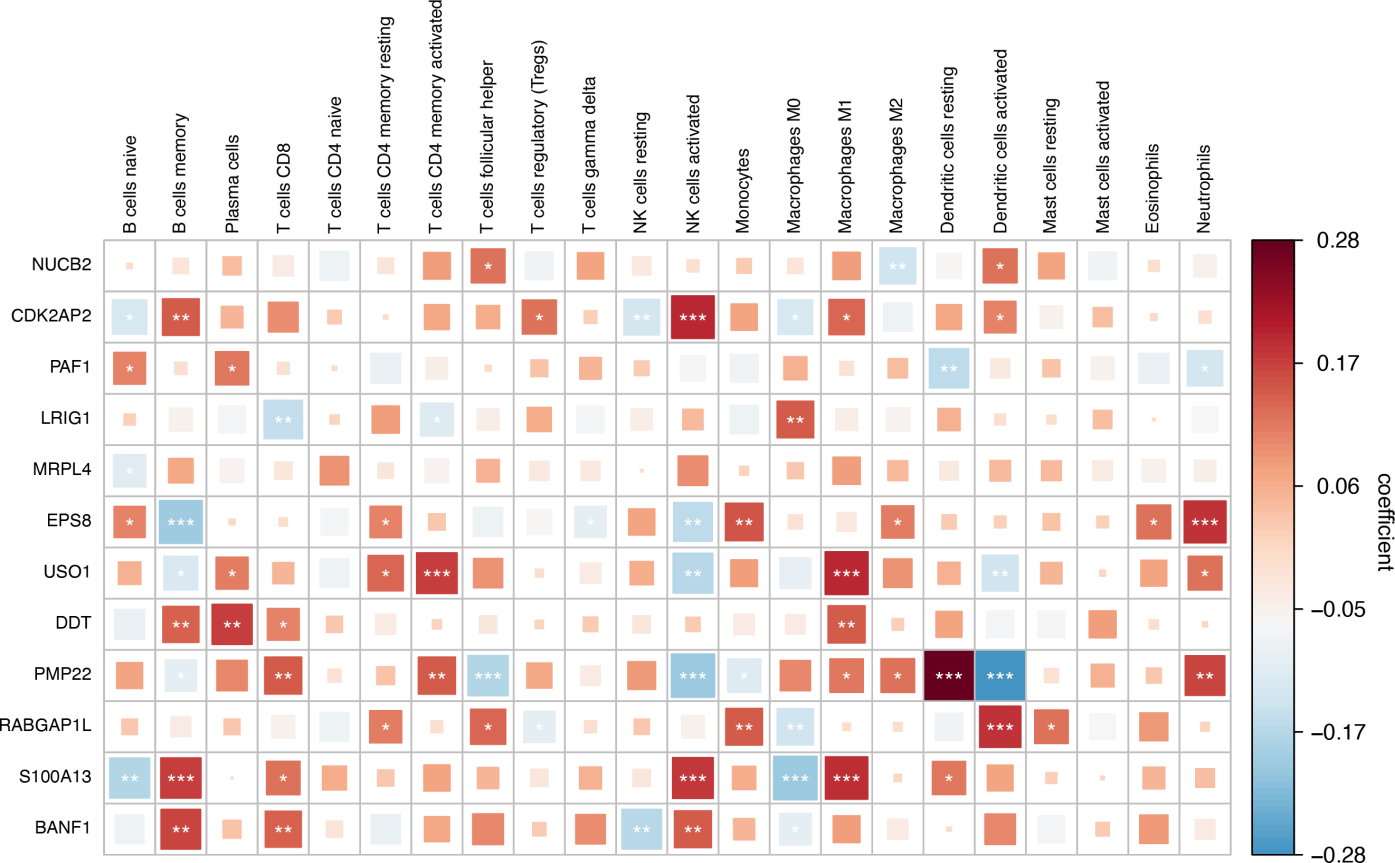
Correlation analysis between key Crotonylation-related genes(CRGs) and immune cells.(*p < 0.05; **p < 0.01; ***p < 0.001). (Spearman’s rank correlation, without applying multiple testing corrections).

To explore immunoregulatory potential of the prognostic genes, we conducted a comprehensive correlation analysis between prognostic genes and immunological profiles. **Figure 10** reveals distinct immune interaction patterns. NUCB2 was significantly correlated with follicular helper T cells and activated Dendritic cells. CDK2AP2 displays multifaceted connections involving regulatory T cells (Tregs), activated dendritic cells, and M2 macrophage. BANF1 demonstrates pronounced associations with memory B cells, CD8^+^ T cells, natural killer cell subsets, and M0 macrophages. LRIG1 have strong links with CD8^+^ T cells, activated CD4^+^T memory cells, and M0 Macrophages. PAF1 exhibited significant correlations with B cells (naive), plasma cells, dendritic cells (resting), and neutrophils. MRPL4 exhibited a significant correlation with naive B cells. EPS8 showed significant correlations with naive B cells, memory B cells, resting CD4^+^ T memory cells, γδ T cells, activated NK cells, monocytes, M2 macrophages, eosinophils, and neutrophils. USO1 was significantly associated with memory B cells, plasma cells, resting and activated CD4^+^ T memory cells, activated NK cells, M1 macrophages, activated dendritic cells, and neutrophils. DDT demonstrated significant correlations with memory B cells, plasma cells, CD8^+^ T cells, and M1 macrophages. PMP22 exhibited significant correlations with memory B cells, CD8^+^ T cells, activated CD4^+^ T memory cells, follicular helper T cells, activated NK cells, monocytes, M1/M2 macrophages, resting/activated dendritic cells, and neutrophils. RABGAP1L showed significant correlations with resting CD4^+^ T memory cells, follicular helper T cells, regulatory T cells (Tregs), monocytes, M0 macrophages, activated dendritic cells, and resting mast cells. S100A13 exhibited significant correlations with naive B cells, memory B cells, CD8+ T cells, activated NK cells, M0/M1 macrophages, and resting dendritic cells. Collectively, these findings indicate that prognostic genes are closely linked to immune regulation.

## Discussion

Crotonylation, a recently identified protein acyl modification characterized by its unique acyl group, not only alters protein structure but also modulates protein stability and activity (8, 43). Research into crotonylation has revealed that crotonylation plays a critical role in various physiological processes, including RNA processing, nucleotide metabolism, and chromatin remodeling (44). Ovarian cancer has high metastatic potential, and the majority of cases are diagnosed at an advanced stage. Furthermore, this malignancy is characterized by a propensity for recurrence and the development of treatment resistance, factors that collectively contribute to a relatively low five-year survival rate (38). Dysregulated crotonylation may cause cell dysfunction and eventually lead to pathological processes such as carcinogenesis and metastasis. A quantitative proteomics study characterized p300-regulated lysine crotonylation, revealing that p300-targeted lysine crotonylation substrates may be involved in cancer development (45). The crotonylation of Lys27 of histone H3 facilitates CRC metastasis (12). These findings suggest that crotonylation could function as a carcinogenic factor, potentially promoting tumor progression; however, further investigation is needed to understand its role in ovarian cancer.

In this study, by integrating single-cell transcriptome and bulk RNA-seq data from patients with ovarian cancer, for the first time, we systematically revealed the regulatory characteristics and prognostic value of protein crotonylation in the progression of ovarian cancer. Initially, we characterized the heterogeneity of the ovarian cancer microenvironment and observed a high degree of immune cell infiltration. Furthermore, leveraging single-cell and bulk transcriptome data, we conducted an extensive analysis of crotonylation levels within this microenvironment. Our findings indicated that crotonylation was abnormally activated in a cell subtype-specific manner in ovarian cancer.

Crotonylation not only serves as a biomarker for molecular stratification but also likely contributes to disease progression by modulating the heterogeneity and plasticity of the TME. According to the EDRN database, 4.5% (20 out of 443) of tumor biomarkers are crotonylated, and 32 crotonylated proteins are associated with tumor-related genes (9). These crotonylated proteins play critical roles in tumorigenesis and tumor development. Numerous studies have demonstrated that crotonylation is decreased in hepatic carcinoma, gastric cancer, and renal carcinoma but increased in thyroid cancer, esophageal cancer, colorectal carcinoma, pancreatic cancer, and lung cancer. These findings suggest that crotonylation may exert effects by regulating distinct cancer-related proteins (9). A previous study revealed that crotonylation facilitates cell invasion through the crotonylated SEPT2-K74-P85α-AKT pathway and that high SEPT2-K74 crotonylation predicts poor prognosis and a high recurrence rate in HCC patients (46). Studies have demonstrated that the level of PGK1 K131cr in advanced breast cancer cells is significantly lower than that in early-stage cells, suggesting that reduced levels of PGK1 K131cr are associated with a poorer prognosis in breast cancer patients (10). A recent study revealed that crotonylated BEX2 interacts with NDP52 and enhances mitophagy to modulate the apoptosis induced by chemotherapeutic agents in non-small cell lung cancer cells (47). p300-mediated lysine crotonylation enhances the expression of HNRNPA1 to promote the proliferation, invasion, and migration of HeLa cells (48). Other crotonylation-regulated proteins, such as SIRT1 (49), SIRT2 (50), and SIRT3 (51), have been confirmed to play regulatory roles in cervical cancer. However, the precise regulatory mechanisms underlying crotonylation have yet to be fully elucidated, necessitating further in-depth investigations.

In this study, we identified 451 key crotonylation-related genes in ovarian cancer and analyzed their potential biological mechanisms. GO analysis revealed that the key crotonylation-related genes in ovarian cancer are associated with several biological processes, including the regulation of cardiac muscle cell apoptosis, aerobic electron transport chain activity, and striated muscle cell apoptosis. These genes are also linked to cellular components such as the respiratory chain complex, mitochondrial respirasome, and respirasome, as well as molecular functions such as NADH dehydrogenase (ubiquinone) activity, NADH dehydrogenase (quinone) activity, and exo-alpha-sialidase activity. KEGG analysis revealed that the key genes are associated with several important pathways, including chemical carcinogenesis mediated by reactive oxygen species (ROS). Crotonylation may also serve as a valuable tool for predicting the prognosis of ovarian cancer patients. Univariate Cox analysis identified 12 prognostic genes in ovarian cancer: BANF1, CDK2AP2, DDT, EPS8, LRIG1, MRPL4, NUCB2, PAF1, PMP22, RABGAP1L, S100A13, and USO1. We employed a combination of 101 machine learning algorithms and constructed predictive models using 10-fold cross-validation, ultimately selecting the random survival forest (RSF) model. We then calculated the risk regression coefficients of the prognostic genes using the Cox proportional hazards regression model and developed a formula for calculating the RS value on the basis of these coefficients. Finally, we validated the prognostic model and confirmed that RSs exhibit excellent predictive performance in patients with ovarian cancer. The RS is an effective quantitative tool for analyzing crotonylation modifications in the clinical diagnosis and treatment of ovarian cancer, guiding the implementation of more targeted therapeutic strategies; its prognostic accuracy for ovarian cancer patients has been validated with data from multiple independent medical centers.

Crotonylation also plays a pivotal role in reshaping the tumor immune microenvironment and modulating the response to immunotherapy. A 2020 study published in Nature reported that the loss of histone lysine crotonylation promotes immunogenic cytosolic dsRNA and dsDNA generation through increased H3K27ac, which stimulates the RNA sensor MDA5 and the DNA sensor cGAS to increase type I interferon signaling, leading to compromised GSC tumorigenic potential and increased CD8^+^ T cell infiltration (52). Lao. et al. reported that GCDH inhibits HCC progression via the crotonylation-induced suppression of the PPP and glycolysis, resulting in HCC cell senescence, and that senescent cells further shape the antitumor microenvironment via the SASP. The GCDH^low^ population is responsive to anti-PD-1 therapy because of the increased presence of PD-1^+^CD8^+^ T cells (53). We investigated the relationships between RS and immunological characteristics, as well as the underlying biological mechanisms. The analysis revealed high-RS groups showed immunosuppressive characteristics, and high-RS was associated with increased stromal activation and enhanced immune evasion potential. Furthermore, we found that step 5 of the tumor immune cycle was significantly activated in the high-RS compared to the low-RS group. The results demonstrated that the expression of nine immune checkpoint genes was significantly different between these two groups, Notably, high-RS groups exhibited upregulated PDL1 and CD40. The above findings indicate that the high RS groups exhibits increased susceptibility to immunotherapy. Pharmacogenomic analysis identified vinblastine with differential sensitivity, providing actionable targets for RS-stratified therapy.

We also conducted a comprehensive analysis of highly variable gene (HVG) scores across different cell subtypes in the high- and low-RS groups, the correlations among HVGs between these subtypes, and the survival rates associated with high and low HVG scores. The results demonstrated that high-RS groups in various cell subtypes, including fibroblasts, endothelial cells, and monocytes within the ovarian cancer microenvironment, exhibited elevated HGV scores, which influenced patient survival rates. These findings further substantiate the critical role of crotonylation in the ovarian cancer microenvironment.

Finally, we examined the expression levels of prognostic genes in ovarian cancer samples. We found that the expression of BANF1, CDK2AP2, DDT, LRIG1, MRPL4, and S100A13 was upregulated in ovarian cancer samples, and the expression of EPS8, NUCB2, PAF1, PMP22, RABGAP1L, and USO1 was upregulated in normal samples. And prognostic genes are closely linked to immune regulation. These may become important markers for predicting the prognosis of ovarian cancer.

Given the crucial role of crotonylation in tumor progression, it holds great potential as a key biomarker for predicting patient prognosis and developing tumor-targeted therapies. However, the role and molecular mechanisms of crotonylation in ovarian cancer remain largely unexplored. This study, for the first time, elucidates the role of crotonylation in the ovarian cancer microenvironment and its potential biological mechanisms. We identified 12 prognostic genes for ovarian cancer, developed a prognostic prediction model based on the crotonylation modification network, and analyzed immune-related characteristics, highly variable gene mutation scores, and drug sensitivity across different prognostic groups. These findings provide a theoretical foundation for prognosis stratification, recurrence monitoring, and the optimization of precision treatment strategies for ovarian cancer. However, this study has several limitations. While single-cell and transcriptome data provide insights into the expression patterns of crotonylation-related genes, there is a lack *in vitro* and *in vivo* functional validation of key targets. Additionally, the prognostic model has not been validated in multicenter cohorts or prospective clinical samples, and there is a need for dynamic monitoring, such as evaluating the correlation between changes in crotonylation scores before and after treatment and therapeutic efficacy. Addressing these gaps will be a key focus of our future research.

## Conclusion

In summary, in our study, We elucidated the significant impact of crotonylation on the ovarian cancer microenvironment and prognosis. We developed and validated a novel prognostic model for ovarian cancer that can serve as a tool for predicting patient outcomes and characterizing the immune microenvironment. These findings enhance our understanding of the role of crotonylation in ovarian cancer and establish a robust framework for developing therapeutic strategies targeting crotonylation.

## Data Availability

The original contributions presented in the study are included in the article/**Supplementary Material**. Further inquiries can be directed to the corresponding author.

## Supplementary material

Supplementary Figure 1 Flowchart representation of the study design.

Supplementary Figure 2 Differential analysis between the high- and low-AUCell score groups. Differential analysis of B cells and plasma cells **(A)**, endothelial cells **(B)**, epithelial cells **(C)**, fibroblasts **(D)**, monocytes **(E)**, SMC myofibroblasts **(F)**, and T cells **(G)** between the high- and low-AUCell score groups.

Supplementary Figure 3 Differential analysis between ovarian cancer(OV) tumor samples and normal samples. **(A)** Volcano plot illustrating the results of the differential expression analysis between OV tumor samples and normal samples in the TCGA database. **(B)** Heatmap of differentially expressed genes between OV tumor samples and normal samples. Red indicates upregulation, and blue indicates downregulation.

Supplementary Figure 4 Gene selection workflow.
