# Supplementary figures and images for: Integrated analysis of single-cell and bulk transcriptomics reveals the prognostic value and underlying mechanisms of crotonylation in ovarian cancer
Source: Front Immunol. 2025 Sep 10;16:1596080. doi: 10.3389/fimmu.2025.1596080 (PMC12457283; doi:10.3389/fimmu.2025.1596080)

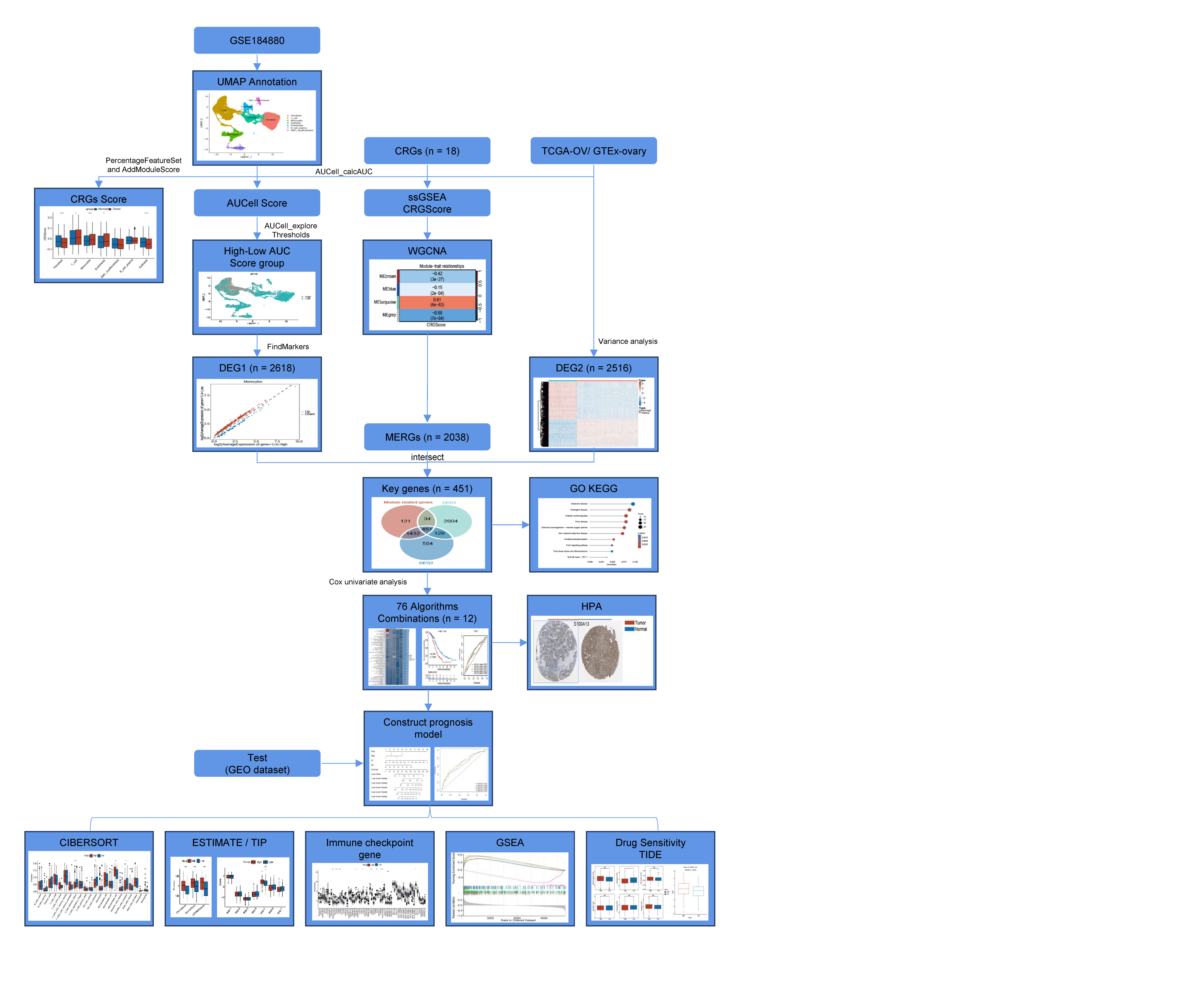

Supplement: Supplementary Figure 1 — Flowchart representation of the study design. [file Image1.jpeg]

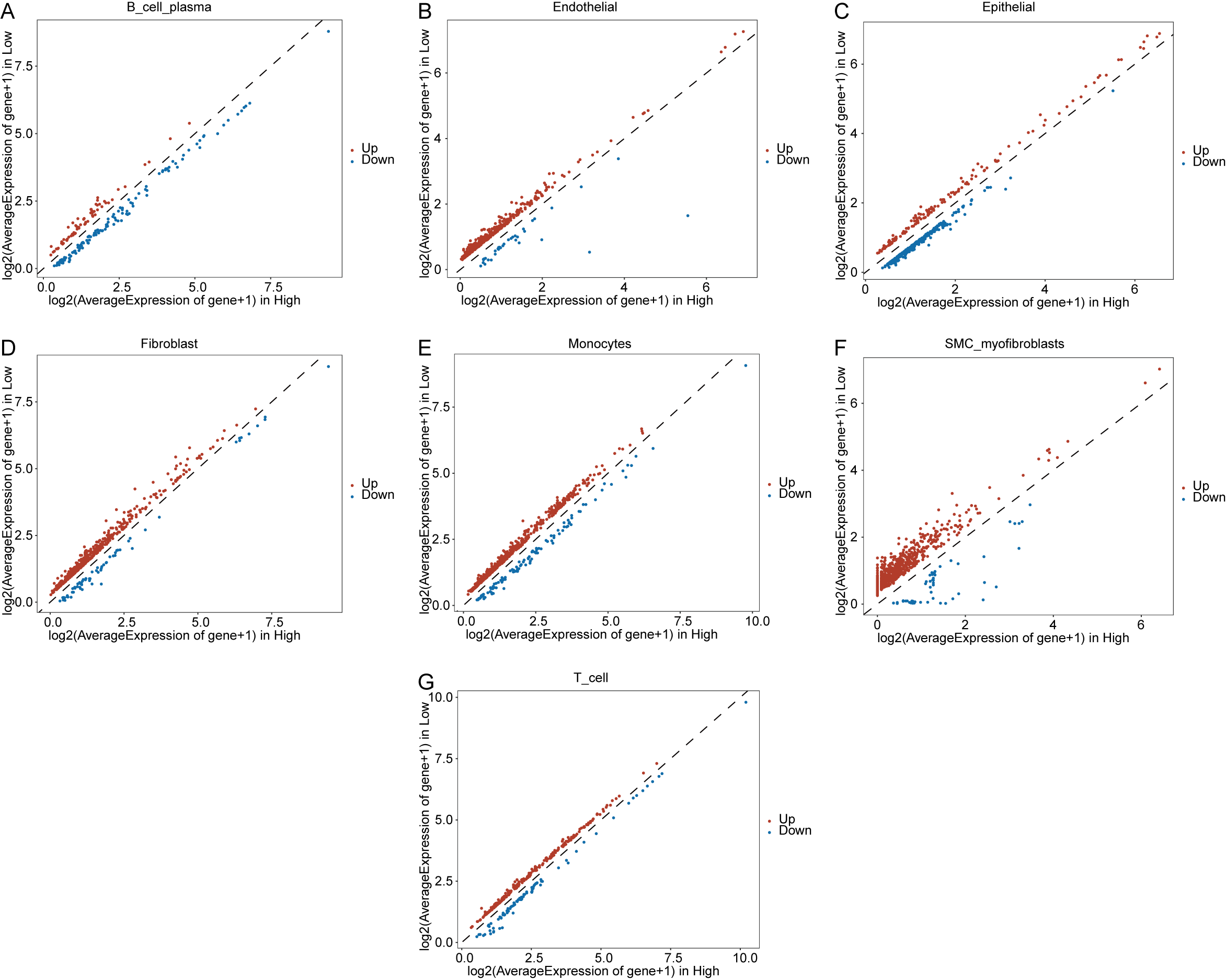

Supplement: Supplementary Figure 2 — Differential analysis between the high- and low-AUCell score groups. Differential analysis of B cells and plasma cells (A), endothelial cells (B), epithelial cells (C), fibroblasts (D), monocytes (E), SMC myofibroblasts (F), and T cells (G) between the high- and low-AUCell score groups. [file Image2.tif]

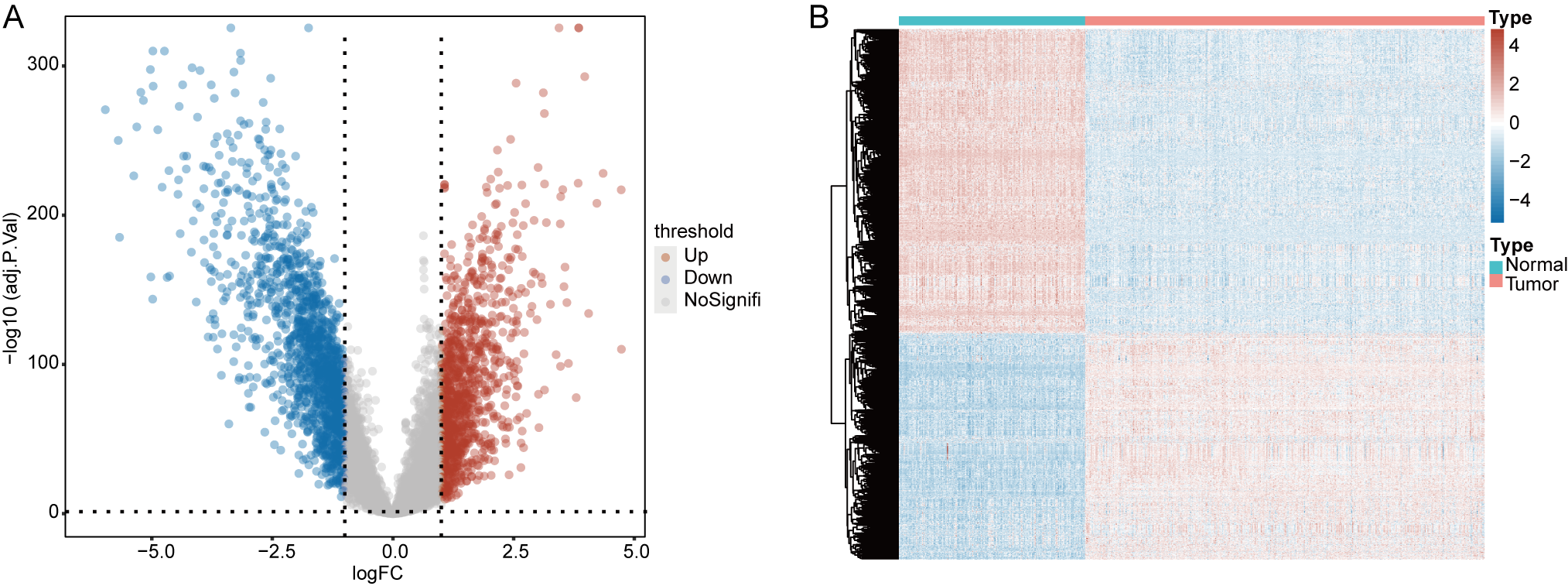

Supplement: Supplementary Figure 3 — Differential analysis between ovarian cancer(OV) tumor samples and normal samples. (A) Volcano plot illustrating the results of the differential expression analysis between OV tumor samples and normal samples in the TCGA database. (B) Heatmap of differentially expressed genes between OV tumor samples and normal samples. Red indicates upregulation, and blue indicates downregulation. [file Image3.tif]

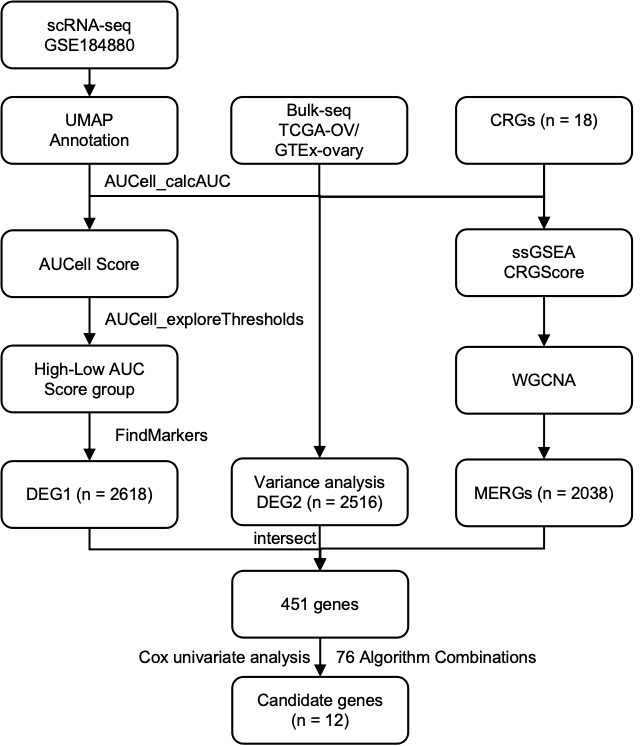

Supplement: Supplementary Figure 4 — Gene selection workflow. [file Image4.tif]
